# Supplementary material for: Affinity-matured homotypic interactions induce spectrum of PfCSP structures that influence protection from malaria infection
Source: Nat Commun. 2023 Jul 28;14:4546. doi: 10.1038/s41467-023-40151-x (PMC10382551; doi:10.1038/s41467-023-40151-x)
Supplement: Supplementary file 1 — Supplementary Information [file 41467_2023_40151_MOESM1_ESM.pdf]

Affinity-matured homotypic interactions induce spectrum of PfCSP-antibody structures that influence protection from malaria infection

Gregory M. Martin[1], Jonathan L. Torres[1], Tossapol Pholcharee[1,7], David Oyen[1,8], Yevel Flores-Garcia[2], Grace Gibson[1], Re'em Moskovitz[1], Nathan Beutler[3], Diana D. Jung[1], Jeffrey Copps[1], Wen-Hsin Lee[1], Gonzalo Gonzalez-Paez[1], Daniel Emerling[5], Randall S. MacGill[6], Emily Locke[6], C. Richter King[6], Fidel Zavala[2], Ian A. Wilson[1,4], Andrew B. Ward[1*]

Affiliations
[1]Department of Integrative Structural and Computational Biology; The Scripps Research Institute; La Jolla, CA 92037; USA.
[2]Department of Molecular Microbiology and Immunology, Malaria Research Institute; Johns Hopkins Bloomberg School of Public Health; Baltimore, MD 21205; USA.
[3]Department of Immunology and Microbiology; The Scripps Research Institute; La Jolla, CA 92037; USA.
[4]The Skaggs Institute for Chemical Biology; The Scripps Research Institute; La Jolla, CA 92037; USA.
[5]Atreca Inc; San Carlos, CA 94070; USA.
[6]PATH's Malaria Vaccine Initiative; Washington, DC 20001; USA.
[7]Present address: Department of Biochemistry, University of Oxford, Oxford OX1 3DR; UK.
[8]Present address: Pfizer Inc; San Diego, CA 92121; USA.

*Correspondence: andrew@scripps.edu

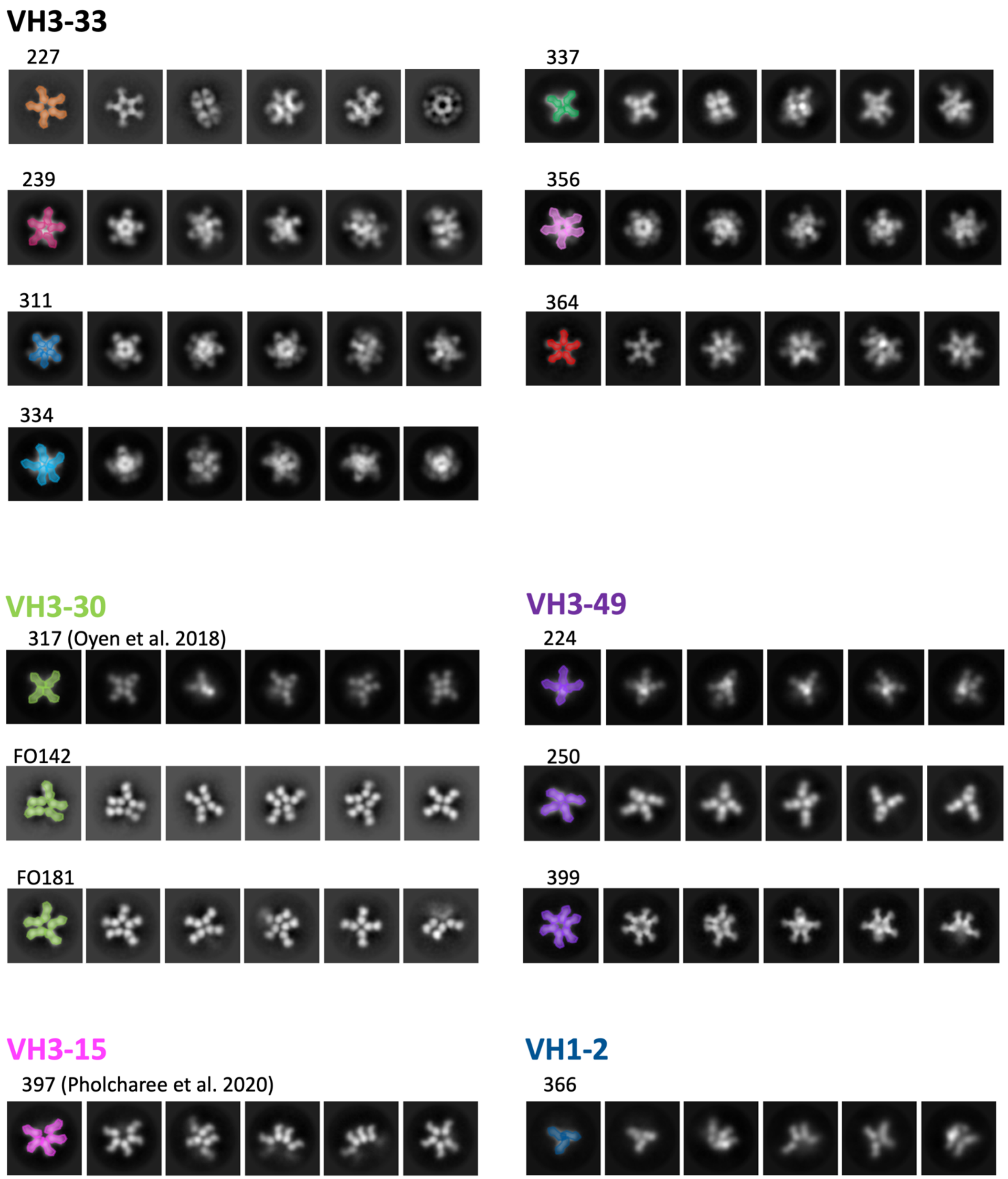

Supplementary Figure 1. **Representative 2D class averages from negative stain EM of anti-NPNA Fabs in complex with rsCSP.** To aid visualization, individual Fabs are colored representing the first (leftmost) class average of each mAb (grey). 2D classes for 317[1] and 397[2] were reprocessed herein from their original, previously published, datasets.

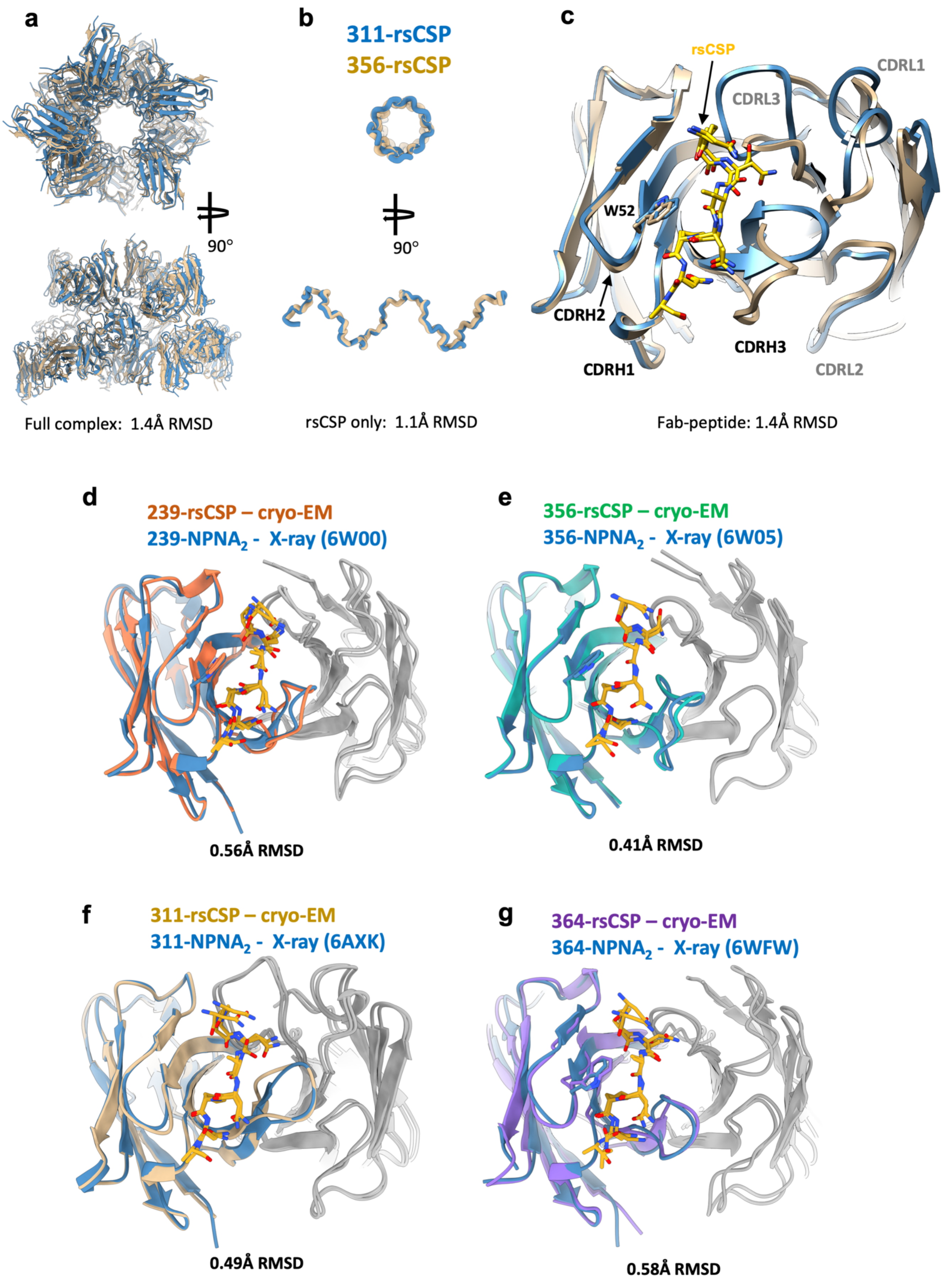
a
b
311-rsCSP
356-rsCSP
c
rsCSP
CDRL1
CDRL3
W52
CDRH2
CDRH1
CDRH3
CDRL2
90°
90°
Full complex: 1.4Å RMSD
rsCSP only: 1.1Å RMSD
Fab-peptide: 1.4Å RMSD
d
239-rsCSP – cryo-EM
239-NPNA$_2$ - X-ray (6W00)
0.56Å RMSD
e
356-rsCSP – cryo-EM
356-NPNA$_2$ - X-ray (6W05)
0.41Å RMSD
f
311-rsCSP – cryo-EM
311-NPNA$_2$ - X-ray (6AXK)
0.49Å RMSD
g
364-rsCSP – cryo-EM
364-NPNA$_2$ - X-ray (6WFW)
0.58Å RMSD

Supplementary Figure 2. **Comparison of cryo-EM structures and previously-published Fab-peptide X-ray structures.** **a-b** Comparison of 311-rsCSP and 356-rsCSP cryo-EM structures from this study. **a** Top (left) and side (right) views of superposition of 311 (blue) and 356 (tan) complexes. **b** Same as in (a), showing only the rsCSP helical spiral to highlight high similarity of CSP helical structures. **c** Superposition of a single Fab from the 311 and 356 cryo-EM structures; CSP is in gold. **d-g** Comparison of cryo-EM structures of Fabs with X-ray crystal structures of corresponding Fabs bound to NPNA peptides. **d** 239-rsCSP cryo-EM and 239-$NPNA_2$ X-ray. **e** 356-rsCSP cryo-EM and 356-$NPNA_2$ X-ray. **f** 311-rsCSP cryo-EM and 311-$NPNA_2$ X-ray. **g** 364-rsCSP cryo-EM and 364-$NPNA_2$ X-ray. **239, 356, 364:** Pholcharee et al. 2021[3]. **311:** Oyen et al. 2017[1].

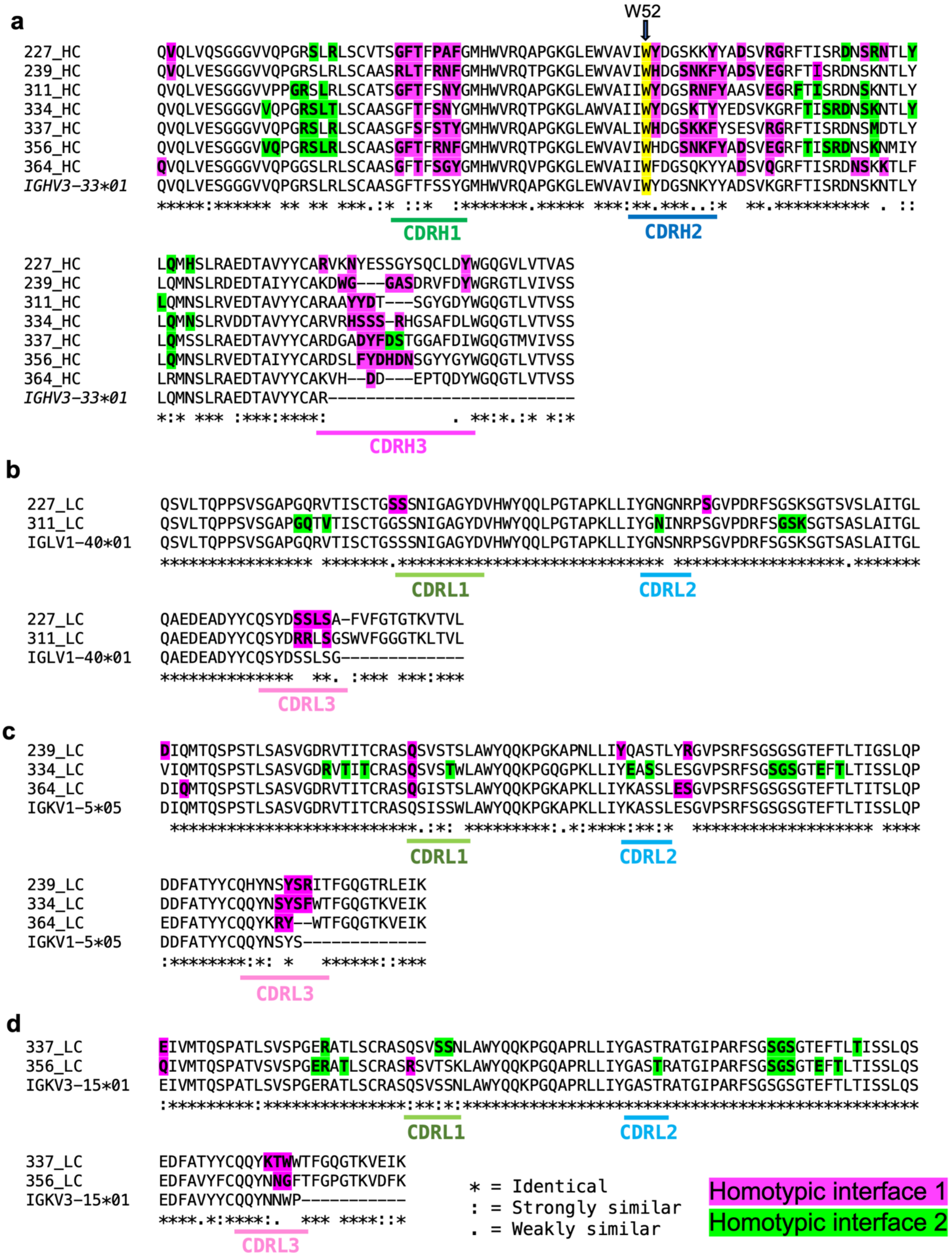
a
W52
227_HC QVQLVQSGGGVVQPGRSLRLSCVTSGFTFPAFGMHWVRQAPGKGLEWVAVIWYDGSKKYYADSVRGRFTISRDNSRNTLY
239_HC QVQLVESGGGVVQPGRSLRLSCAASRLTFRNFGMHWVRQTPGKGLEWVAVIWHDGSNKFYADSVEGRFTISRDNSKNTLY
311_HC QVQLVESGGGVVPPGRSLRLSCATSGFTFSNYGMHWVRQAPGKGLEWVAIIWYDGSRNFYAASVEGRFTISRDNSKNTLY
334_HC QVQLVESGGGVVQPGRSLTLSCAASGFTFSNYGMHWVRQTPGKGLAWVAIIWYDGSKTYYEDSVKGRFTISRDNSKNTLY
337_HC QVQLVESGGGVVQPGRSLRLSCAASGFSFSTYGMHWVRQAPGKGLEWVALIWHDGSKKFYSESVRGRFTISRDNSMDTLY
356_HC QVQLVESGGGVVQPGRSLRLSCAASGFTFRNFGMHWVRQTPGKGLEWVAVIWHDGSNKFYADSVEGRFTISRDNSKNMIY
364_HC QVQLVESGGGVVQPGGSLRLSCAASGFTFSGYGMHWVRQVPGKGLEWVAIIWFDGSQKYYADSVQGRFTISRDNSKKTLF
IGHV3-33*01 QVQLVESGGGVVQPGRSLRLSCAASGFTFSSYGMHWVRQAPGKGLEWVAVIWYDGSNKYYADSVKGRFTISRDNSKNTLY
*****:****** ** ** ***.:* ::* :*******.***** ***:**.***..:* **.********** . ::
CDRH1
CDRH2
227_HC LQMHSLRAEDTAVYYCARVKNYESSGYSQCLDYWGQGVLVTVAS
239_HC LQMNSLRDEDTAIYYCAKDWG---GASDRVFDYWGRGTLVIVSS
311_HC LQMNSLRVEDTAVYYCARAAYYDT---SGYGDYWGQGTLVTVSS
334_HC LQMNSLRVDDTAVYYCARVRHSSS-RHGSAFDLWGQGTLVTVSS
337_HC LQMSSLRAEDTAVYYCARDGADYFDSTGGAFDIWGQGTMVIVSS
356_HC LQMNSLRVEDTAIYYCARDSLFYDHDNSGYYGYWGQGTLVTVSS
364_HC LRMNSLRAEDTAVYYCAKVH--DD---EPTQDYWGQGTLVTVSS
IGHV3-33*01 LQMNSLRAEDTAVYYCAR--------------------------
: *** :***:****: . **:*.:* *:*
CDRH3
b
227_LC QSVLTQPPSVSGAPGQRVTISCTGSSSNIGAGYDVHWYQQLPGTAPKLLIYGNGNRPSGVPDRFSGSKSGTSVSLAITGL
311_LC QSVLTQPPSVSGAPGQTVTISCTGGSSNIGAGYDVHWYQQLPGTAPKLLIYGNINRPSGVPDRFSGSKSGTSASLAITGL
IGLV1-40*01 QSVLTQPPSVSGAPGQRVTISCTGSSSNIGAGYDVHWYQQLPGTAPKLLIYGNSNRPSGVPDRFSGSKSGTSASLAITGL
**************** *******.**************************** ******************.*******
CDRL1
CDRL2
227_LC QAEDEADYYCQSYDSSLSA-FVFGTGTKVTVL
311_LC QAEDEADYYCQSYDRRLSGSWVFGGGTKLTVL
IGLV1-40*01 QAEDEADYYCQSYDSSLSG------------
************** **. :*** ***:***
CDRL3
c
239_LC DIQMTQSPSTLSASVGDRVTITCRASQSVSTSLAWYQQKPGKAPNLLIYQASTLYRGVPSRFSGSGSGTEFTLTIGSLQP
334_LC VIQMTQSPSTLSASVGDRVTITCRASQSVSTWLAWYQQKPGQGPKLLIYEASSLESGVPSRFSGSGSGTEFTLTISSLQP
364_LC DIQMTQSPSTLSASVGDRVTITCRASQGISTSLAWYQQKPGKAPKLLIYKASSLESGVPSRFSGSGSGTEFTLTITSLQP
IGKV1-5*05 DIQMTQSPSTLSASVGDRVTITCRASQSISSWLAWYQQKPGKAPKLLIYKASSLESGVPSRFSGSGSGTEFTLTISSLQP
**************************.:*: *********:.*:****:**:* ******************* ****
CDRL1
CDRL2
239_LC DDFATYYCQHYNSYSRITFGQGTRLEIK
334_LC DDFATYYCQQYNSYSFWTFGQGTKVEIK
364_LC EDFATYYCQQYKRY--WTFGQGTKVEIK
IGKV1-5*05 DDFATYYCQQYNSYS-------------
:********:*: * ******::***
CDRL3
d
337_LC EIVMTQSPATLSVSPGERATLSCRASQSVSSNLAWYQQKPGQAPRLLIYGASTRATGIPARFSGSGSGTEFTLTISSLQS
356_LC QIVMTQSPATVSVSPGERATLSCRASRSVTSKLAWYQQKPGQAPRLLIYGASTRATGIPARFSGSGSGTEFTLTISSLQS
IGKV3-15*01 EIVMTQSPATLSVSPGERATLSCRASQSVSSNLAWYQQKPGQAPRLLIYGASTRATGIPARFSGSGSGTEFTLTISSLQS
:*********:***************:**:*:***
CDRL1
CDRL2
337_LC EDFATYYCQQYKTWWTFGQGTKVEIK
356_LC EDFAVYFCQQYNNGFTFGPGTKVDFK
IGKV3-15*01 EDFAVYYCQQYNNWP-----------
****.*:****:. *** ****::*
CDRL3
* = Identical
: = Strongly similar
. = Weakly similar
Homotypic interface 1
Homotypic interface 2

Supplementary Figure 3. **Multiple sequence alignment of heavy chain and light chain variable regions of the seven *IGHV3-33* mAbs in this study with inferred germline genes.** **a** Heavy chains aligned to *IGHV3-33*01*. **b** *IGLV1-40* light chains. **c** *IGKV1-5* light chains. **d** *IGKV3-15* light chains.

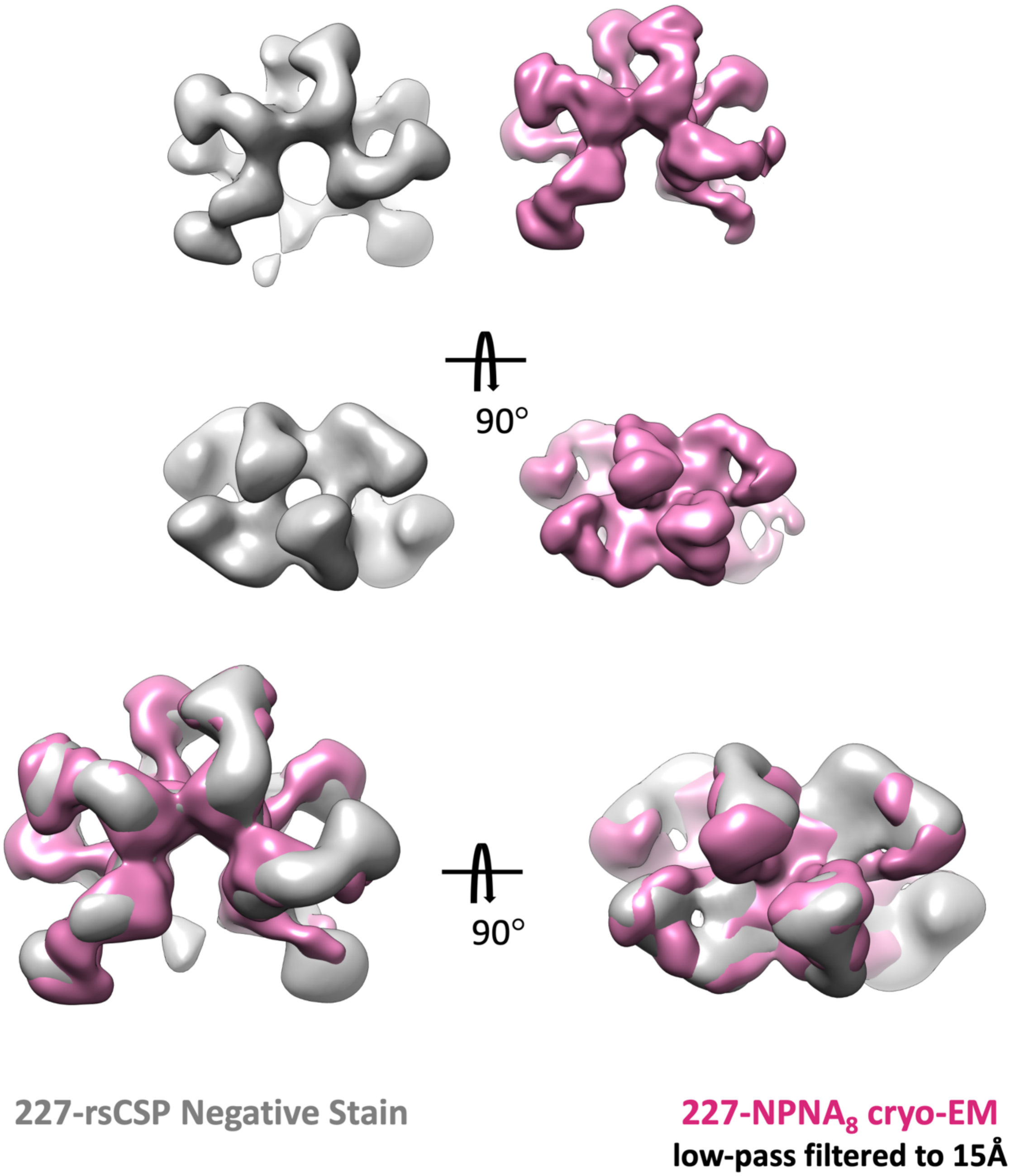

Supplementary Figure 4. **227-NPNA$_8$ and 227-rsCSP structures are nearly identical.** Comparison of negative stain reconstruction of 227 Fab in complex with rsCSP (gray) and cryo-EM structure of 227 Fab in complex with NPNA$_8$ peptide (violet).

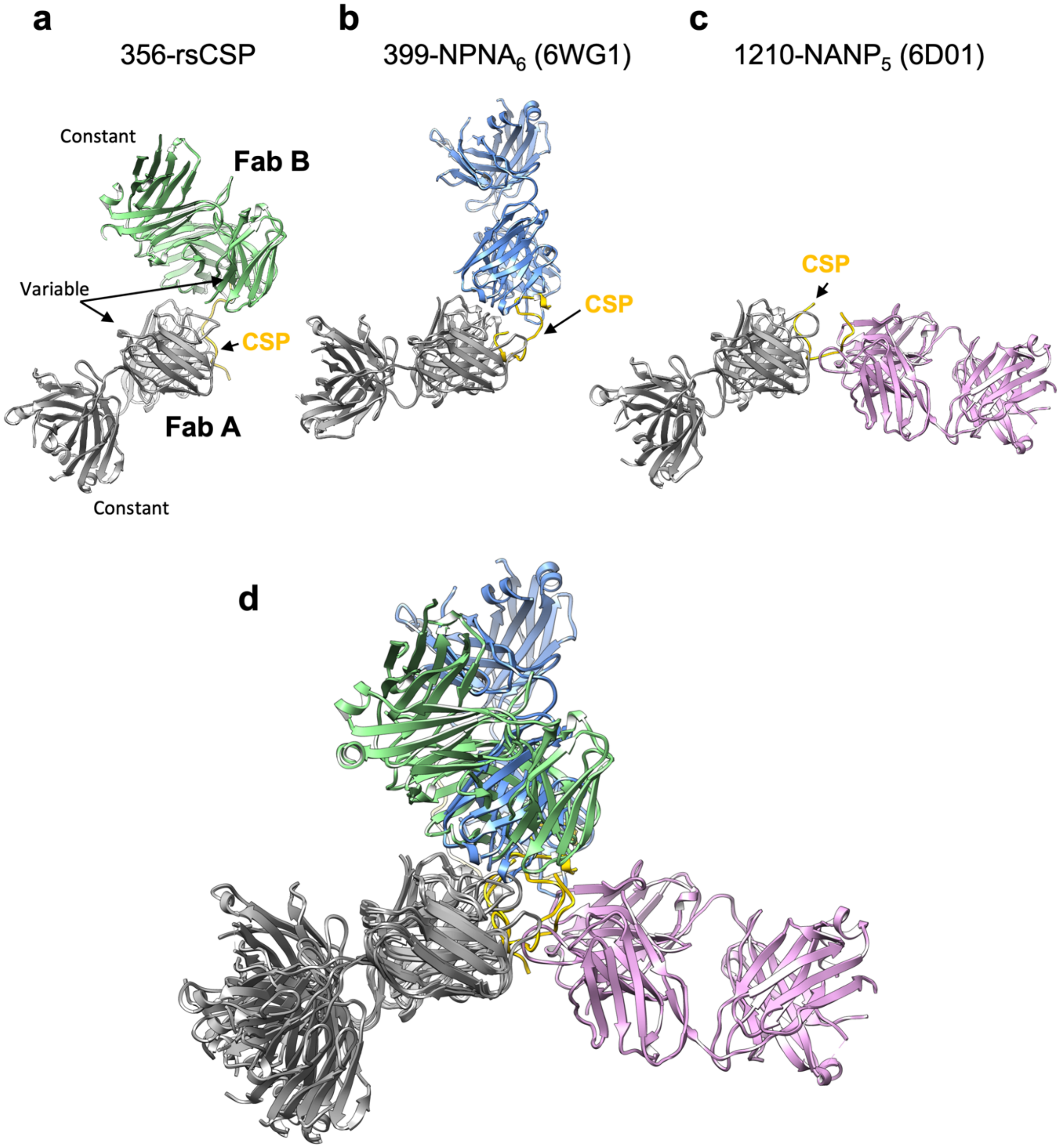

Supplementary Figure 5. **Comparison of homotypic antibody-antibody binding observed in anti-NPNA mAbs. Fabs A of 399 and 1210 were aligned to Fab A of 356 cryo-EM structure for comparison of the position of Fab B relative to Fab A.** **a** Two adjacent Fabs from 356-rsCSP cryo-EM structure, which result in an asymmetric, edge-to-edge interaction. **b** 399-NPNA$_6$ X-ray crystal structure. Note in this structure, nearly all homotypic interactions derive from the heavy chain, and produce a fully symmetric, head-to-head interface. **c** 1210-NANP$_5$ X-ray structure, which gives rise to an asymmetric, head-to-head homotypic interface. **d** Superposition of each structure.

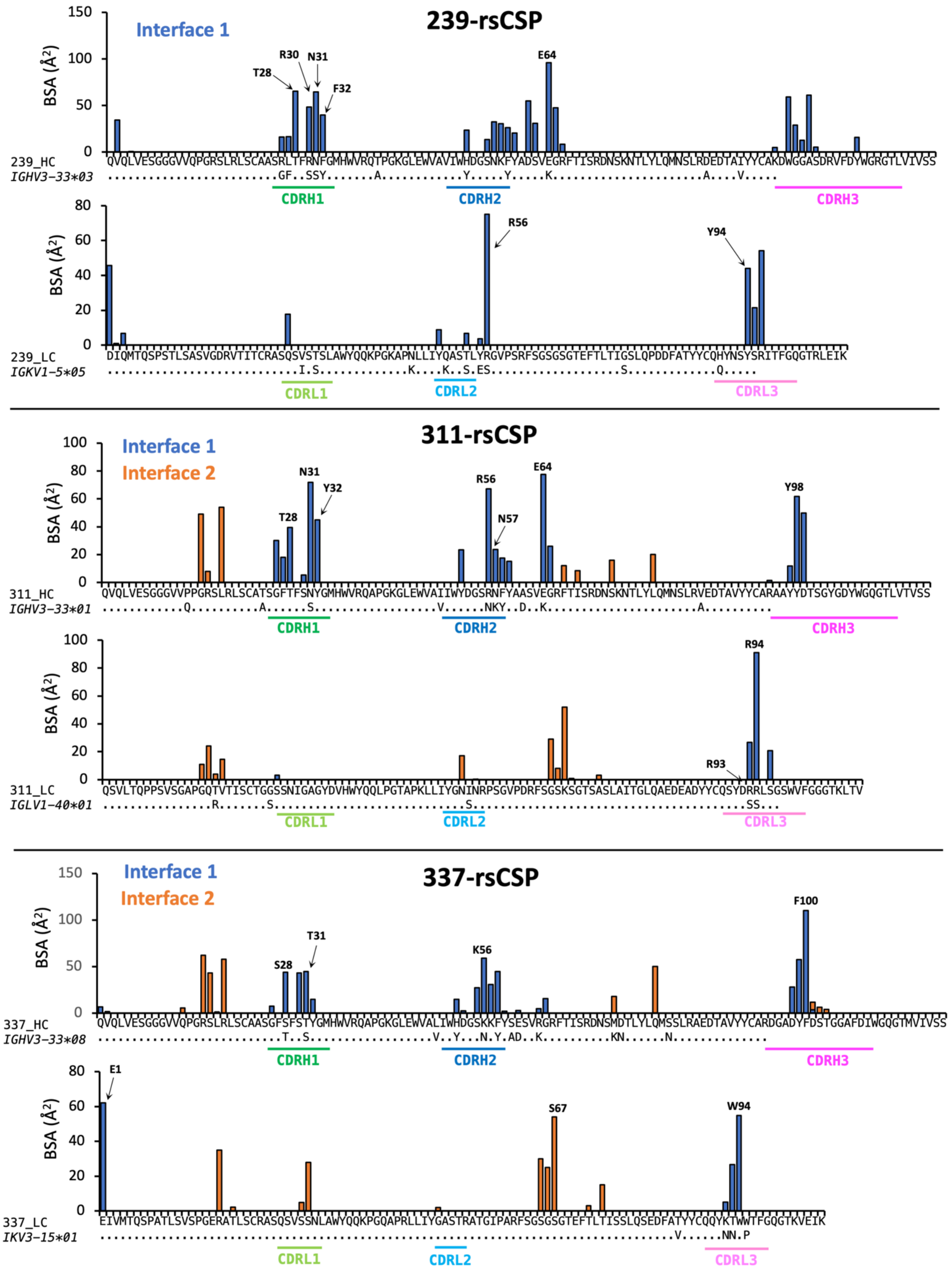

Supplementary Figure 6. **Buried surface area plots for primary and secondary homotypic interfaces for 239, 311, and 337 Fab-rsCSP cryo-EM structures**. Key residues are labelled according to Kabat numbering system.

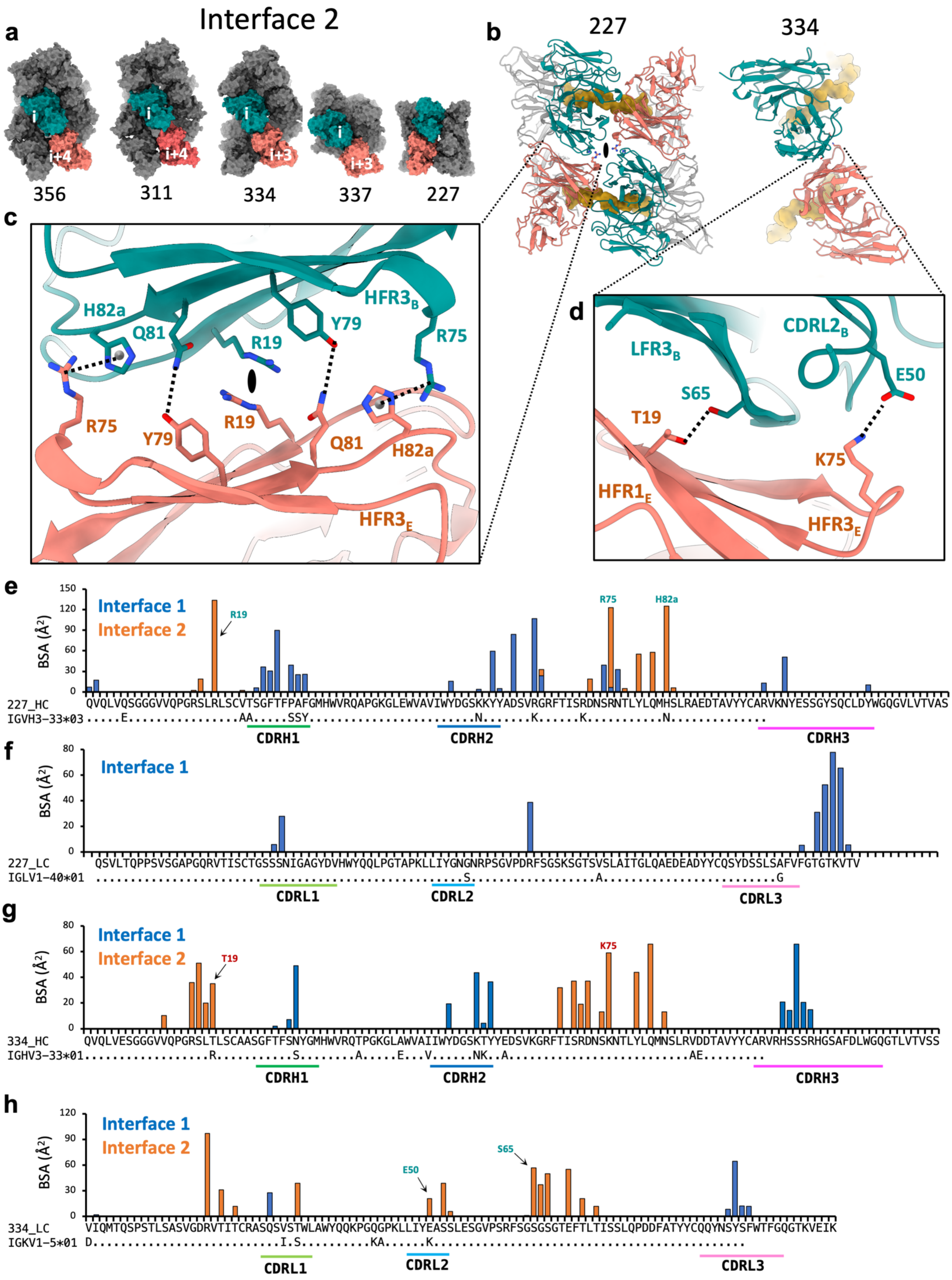

Interface 2
a
356
311
334
337
227
i
i+4
i+3
b
227
334
c
H82a
Q81
R19
Y79
HFR3B
R75
R75
Y79
R19
Q81
H82a
HFR3E
d
LFR3B
CDRL2B
S65
E50
T19
K75
HFR1E
HFR3E
e
Interface 1
Interface 2
BSA (Å²)
R19
R75
H82a
227_HC QVQLVQSGGGVVQPGRSLRLSCVTSGFTFPAFGMHWVRQAPGKGLEWVAVIWYDGSKKYYADSVRGRFTISRDNSRNTLYLQMHSLRAEDTAVYYCARVKNYESSGYSQCLDYWGQGVLVTVAS
IGVH3-33*03
CDRH1
CDRH2
CDRH3
f
Interface 1
BSA (Å²)
227_LC QSVLTQPPSVSGAPGQRVTISCTGSSSNIGAGYDVHWYQQLPGTAPKLLIYGNGNRPSGVPDRFSGSKSGTSVSLAITGLQAEDEADYYCQSYDSSLSAFVFGTGTKVTV
IGLV1-40*01
CDRL1
CDRL2
CDRL3
g
Interface 1
Interface 2
BSA (Å²)
T19
K75
334_HC QVQLVESGGGVVQPGRSLTLSCAASGFTFSNYGMHWVRQTPGKGLAWVAIIWYDGSKTYYEDSVKGRFTISRDNSKNTLYLQMNSLRVDDTAVYYCARVRHSSSRHGSAFDLWGQGTLVTVSS
IGHV3-33*01
CDRH1
CDRH2
CDRH3
h
Interface 1
Interface 2
BSA (Å²)
E50
S65
334_LC VIQMTQSPSTLSASVGDRVTITCRASQSVSTWLAWYQQKPGQGPKLLIYEASSLESGVPSRFSGSGSGTEFTLTISSLQPDDFATYYCQQYNSYSFWTFGQGTKVEIK
IGKV1-5*01
CDRL1
CDRL2
CDRL3

Supplementary Figure 7. **Structure of the secondary homotypic interface (Interface 2).** **a** Surface representation of the five structures in the panel that contain a secondary homotypic interface, which exists between Fabs separated by one helical turn (Fab i and i+x). **b** Cartoon representation of 227 and 334, highlighting the secondary interface. rsCSP is shown as a gold surface. Only Fabs i and i+3 are shown in 334 structure for clarity. The elongated oval in 227 is the overall C2 symmetry axis of the complex. **c** Details of Interface 2 in 227, which is symmetric and mediated exclusively by heavy chain framework regions (FR). Local C2 axis is indicated with black oval. **d** Details of Interface 2 in 334 that is mediated by light chain framework region 3 (LFR3) and CDRL2 with the heavy chain framework regions 1 and 3 (HFR1 and 3). **e-h** BSA plots of homotypic interface 1 and 2 for 227 (e,f) and 334 (g,h).

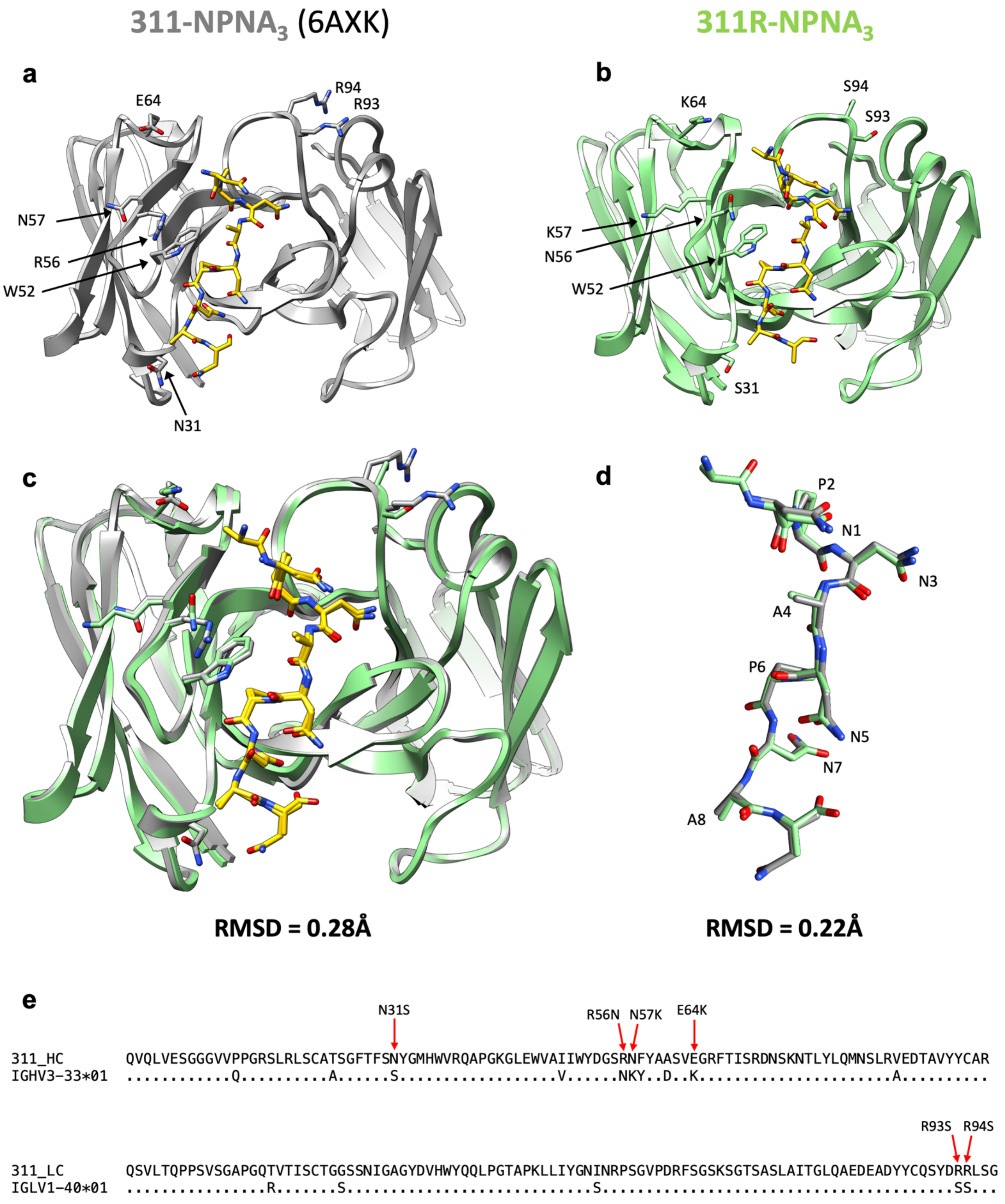

311-NPNA3 (6AXK)
311R-NPNA3
a
E64
R94
R93
N57
R56
W52
N31
b
K64
S94
S93
K57
N56
W52
S31
c
d
P2
N1
N3
A4
P6
N5
N7
A8
RMSD = 0.28Å
RMSD = 0.22Å
e
N31S
R56N
N57K
E64K
311_HC QVQLVESGGGVVPPGRSLRLSCATSGFTFSNYGMHWVRQAPGKGLEWVAIIWYDGSRNFYAASVEGRFTISRDNSKNTLYLQMNSLRVEDTAVYYCAR
IGHV3-33*01 ............Q..........A......S..................V.....NKY..D..K......................A...........
R93S
R94S
311_LC QSVLTQPPSVSGAPGQTVTISCTGGSSNIGAGYDVHWYQQLPGTAPKLLIYGNINRPSGVPDRFSGSKSGTSASLAITGLQAEDEADYYCQSYDRRLSG
IGLV1-40*01 ................R.......S............................S..SS...

Supplementary Figure 8. **Comparison of 311 and 311R X-ray structures.** **a** 311-$NPNA_3$ crystal structure (PDB 6AXK). Residues that were mutated are shown, with W52 shown for reference. CSP is in gold. **b** 311R-$NPNA_3$ X-ray structure (this study). **c** Superposition of 311 and 311R structures. **d** Superposition of CSP structures from 6AXK (gray) and 311R (green). **e** Sequence alignment of 311 heavy and light chain variable regions with respective germline sequences. Residues mutated in 311R are shown with arrows.

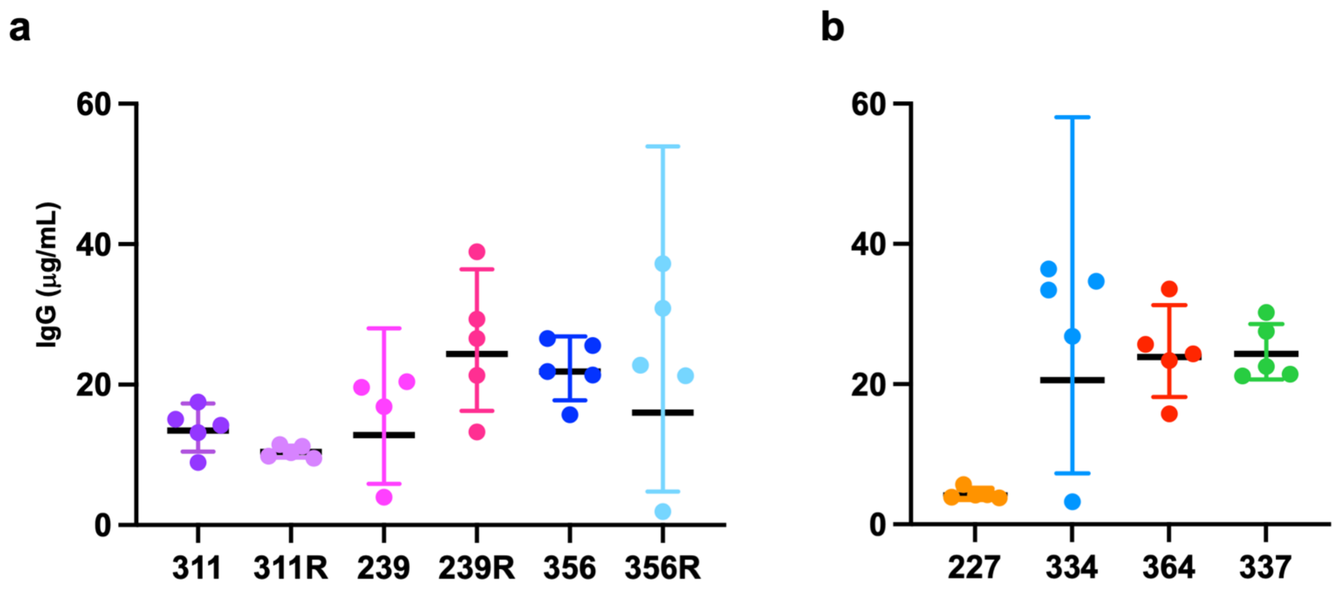

Supplementary Figure 9. **Circulating concentrations of passively administered IgGs in mice, measured at time of challenge.** Serum titers were measured by anti-human IgG ELISA, and calculated based on a standard curve. **a** WT and germline reverted 311, 239, and 356 IgG. **b** WT IgGs of 227, 334, 337, and 364. For each group (antibody), N=5 mice; data points represent individual mice. Error bars represent the geometric mean and SD.

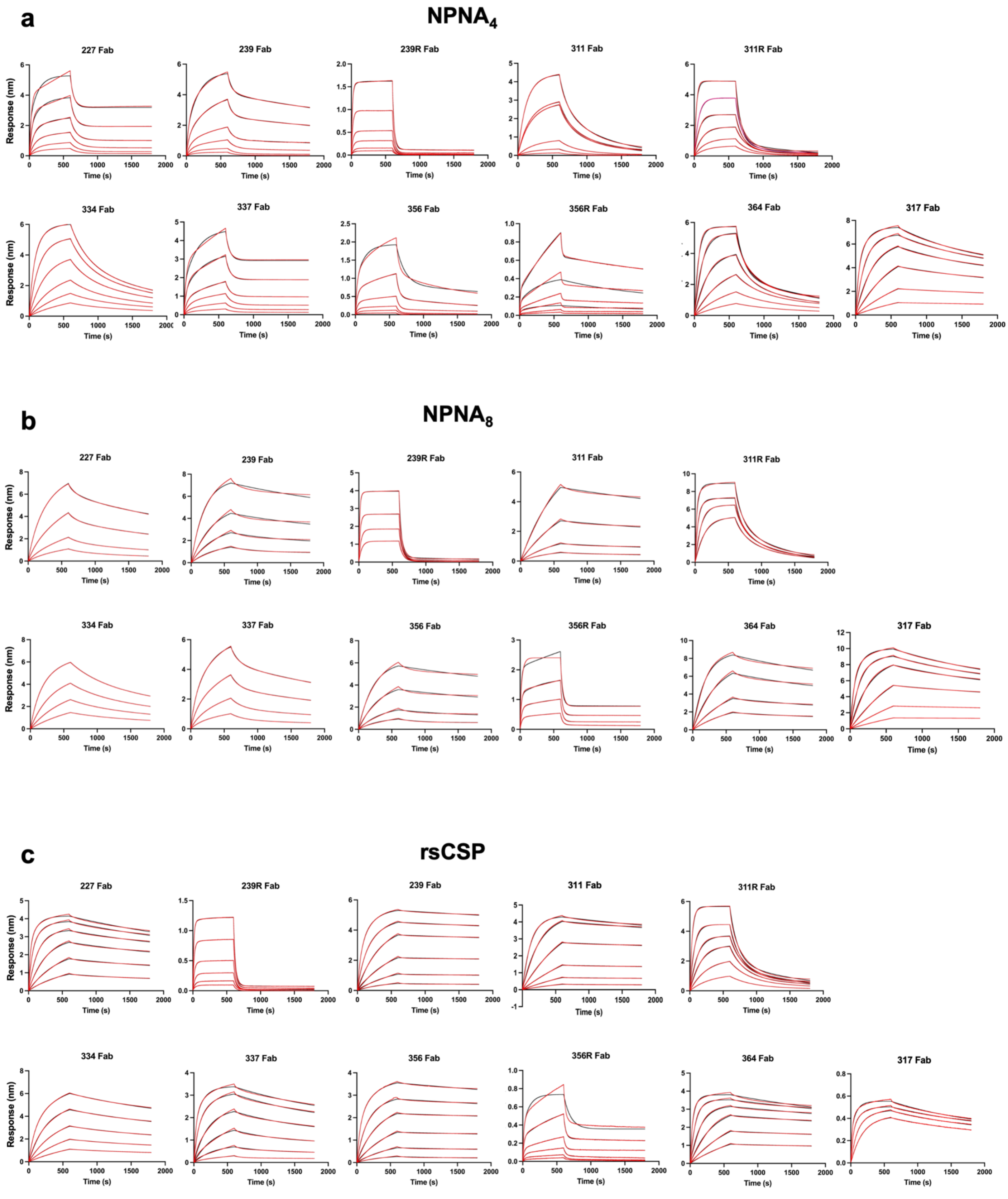
a
NPNA4
227 Fab
239 Fab
239R Fab
311 Fab
311R Fab
334 Fab
337 Fab
356 Fab
356R Fab
364 Fab
317 Fab
b
NPNA8
227 Fab
239 Fab
239R Fab
311 Fab
311R Fab
334 Fab
337 Fab
356 Fab
356R Fab
364 Fab
317 Fab
c
rsCSP
227 Fab
239R Fab
239 Fab
311 Fab
311R Fab
334 Fab
337 Fab
356 Fab
356R Fab
364 Fab
317 Fab
Response (nm)
Time (s)

Supplementary Figure 10. **Representative BLI traces for binding of each Fab to PfCSP peptides.** Binding curves (black) were fit with a 2:1 kinetic model in the Octet Data Analysis software (red). Each Fab was titrated over the following concentrations (nM): 200, 100, 50, 25, 12.5, 6.25. Each binding reaction was run in duplicate. Only traces with $R^2 \geq 0.98$ are shown and were used for affinity calculations, which were averaged across at least four concentrations. **a** $NPNA_4$ peptide. **b** $NPNA_8$ peptide. **c** rsCSP (15 NPNA repeats).

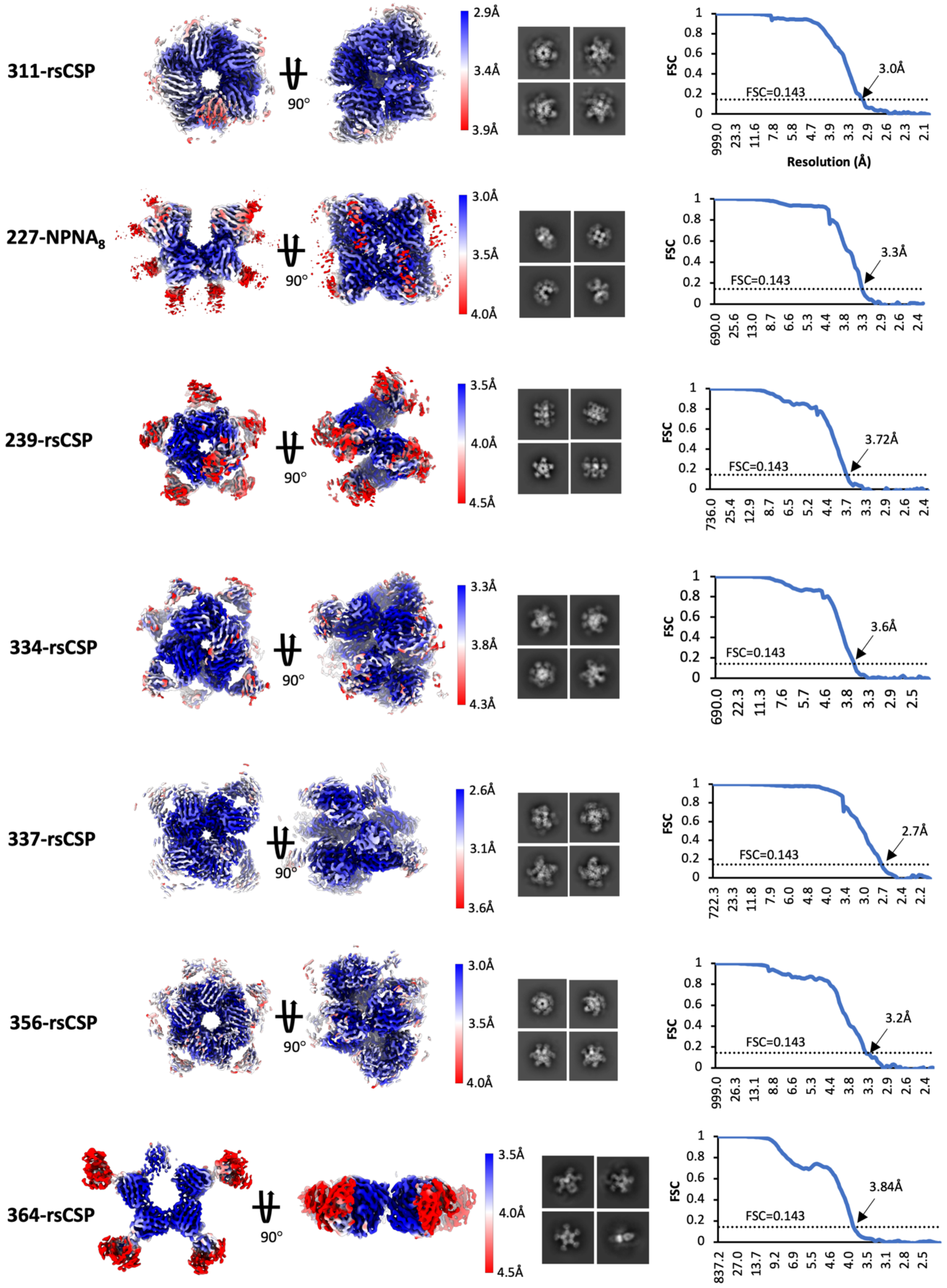

311-rsCSP
90°
2.9Å
3.4Å
3.9Å
FSC
3.0Å
FSC=0.143
999.0 23.3 11.6 7.8 5.8 4.7 3.9 3.3 2.9 2.6 2.3 2.1
Resolution (Å)
227-NPNA8
90°
3.0Å
3.5Å
4.0Å
FSC
3.3Å
FSC=0.143
690.0 25.6 13.0 8.7 6.6 5.3 4.4 3.8 3.3 2.9 2.6 2.4
239-rsCSP
90°
3.5Å
4.0Å
4.5Å
FSC
3.72Å
FSC=0.143
736.0 25.4 12.9 8.7 6.5 5.2 4.4 3.7 3.3 2.9 2.6 2.4
334-rsCSP
90°
3.3Å
3.8Å
4.3Å
FSC
3.6Å
FSC=0.143
690.0 22.3 11.3 7.6 5.7 4.6 3.8 3.3 2.9 2.5
337-rsCSP
90°
2.6Å
3.1Å
3.6Å
FSC
2.7Å
FSC=0.143
722.3 23.3 11.8 7.9 6.0 4.8 4.0 3.4 3.0 2.7 2.4 2.2
356-rsCSP
90°
3.0Å
3.5Å
4.0Å
FSC
3.2Å
FSC=0.143
999.0 26.3 13.1 8.8 6.6 5.3 4.4 3.8 3.3 2.9 2.6 2.4
364-rsCSP
90°
3.5Å
4.0Å
4.5Å
FSC
3.84Å
FSC=0.143
837.2 27.0 13.7 9.2 6.9 5.5 4.6 4.0 3.5 3.1 2.8 2.5

Supplementary Figure 11. **Cryo-EM reconstructions of the seven *IGHV3-33* mAbs in this study. Left column**: top and side views of final EM map, colored by local resolution; color key at right. **Middle column:** representative 2D class averages. **Right column:** Fourier shell correlation for the corrected (noise-substituted) reconstruction. Note that for 311-rsCSP, cryo-EM data originally published in Oyen et al. (2018) were reprocessed here with cryoSPARCv2 and RELION3.0.

Supplementary Table 1. Cryo-EM data collection parameters and model statistics

	**227-NPNA$_8$**	**239-rsCSP**	**311-rsCSP**	**334-rsCSP**	**337-rsCSP**	**356-rsCSP**	**364-rsCSP**
	PDB: 8DYT	PDB: 8DYW	PDB: 8DYX	PDB: 8DYY	PDB: 8DZ3	PDB: 8DZ4	PDB: 8DZ5
	EMDB: 27781	EMDB: 27784	EMDB: 27785	EMDB: 27786	EMDB: 27787	EMDB: 27788	EMDB: 27789
**Data Collection**							
Microscope	Talos Arctica	Talos Arctica	Titan Krios	Talos Arctica	Titan Krios	Talos Arctica	Talos Arctica
Detector	K2 Summit	K2 Summit	K2 Summit	K2 Summit	K2 Summit	K2 Summit	K2 Summit
Voltage (kV)	200	200	300	200	300	200	200
Pixel Size (Å)	1.15	1.15	1.03	1.15	1.03	1.15	1.15
Defocus range (μm)	-0.5 to -2.0	-1.0 to -2.2	-0.5 to -2.5	-1.0 to -2.2	-0.9 to -2.1	-1.0 to -2.2	-1.0 to -2.2
Total dose (e^-/Å^2)	50	50	62	50	50	50	50
Dose rate (e^-/Å^2/sec)	5.3	5.3	5.2	5.3	5.7	5.3	5.3
Frames per exposure	50	50	48	50	50	50	50
**Data Processing**							
Total micrographs	1595	1610	1497	2759	966	696	2049
Particle images in map	70,120	227,439	399,027	458,706	461,179	189,641	723,314
Symmetry imposed	C2	C1	C1	C1	C1	C1	C1
Map res. (FSC=0.143; Å)	3.3	3.72	3.01	3.62	2.68	3.2	3.84
Map sharpening B-factor (Å^2)	-122.5	-140.6	-54.2	-131.5	-76.6	-21.1	-187
Processing software	cryoSPARC v3.3	cryoSPARC v3.3	RELION3.0	cryoSPARC v3.3	cryoSPARC v3.3	cryoSPARC v3.3	cryoSPARC v3.3
**Model Refinement**							
No. atoms in deposited model	14,586	18,495	20,030	16,499	12,727	20,312	8950
Chains total	18	21	23	19	15	23	11
Residues (protein)	1920	2370	2653	2133	1659	2618	1155
RMS Deviations							
Bond lengths (Å)	0.007	0.006	0.003	0.011	0.003	0.002	0.003
Bond angles (°)	0.674	0.846	0.567	0.957	0.585	0.55	0.71
Validation							
Molprobity score	1.8	1.93	1.37	1.92	2.0	1.53	2.0
Clashscore	8.3	14	4.74	10	7.0	6.7	12.5
EMRinger score	4.67	2.57	4.54	3.28	4.1	4.16	2.2
Poor rotamers (%)	0.45	0.35	0.33	0.28	3.4	0.09	0.73
Ramachandran plot							
Favored (%)	94.9	95.9	97.3	94.1	96.5	97.1	94.6
Allowed (%)	5.1	3.7	2.7	5.9	3.5	2.9	5.4
Outliers (%)	0	0.4	0	0	0	0	0
Average B-factor	56.9	80.5	67.4	41.1	55.1	58.4	69.2

Supplementary Table 2. Buried surface area (BSA) calculations

	Full Epitope BSA ($Å^2$)		Homotypic Interface BSA ($Å^2$)	
**mAb**	**Fab BSA**	**CSP BSA**	**Interface 1**	**Interface 2**
227	837	911	1042	552
239	964	1044	1139	n/a
311	510	662	727	331
334	1058	1127	425	950
337	941	1110	714	455
356	888	975	899	674
364	878	955	738	n/a

Supplementary Table 2. **Buried surface areas (BSA) for individual Fabs bound to rsCSP, and for the two homotypic interfaces in the rsCSP complex.**

Table S3. Homotypic contacts in 227-NPNA$_8$ cryo-EM structure

Interface	Chain Fab 1	Residue 1	Position 1	Atom	Chain Fab 2	Residue 2	Position 2	Distance (Å)	Predicted interaction
1	H	GLN	1	NE2-CD2	D	LEU	95	4.1	Van-der-Waals
1	H	SER	25	C-OD2	C	ASP	61	3.3	Van-der-Waals
1	H	GLY	26	N-OD2	C	ASP	61	2.4	H-bond
1	H	GLY	26	N-OD1	C	ASP	61	4.4	H-bond
1	H	GLY	26	O-CG	D	LEU	95	3.6	Van-der-Waals
1	H	PHE	27	N-OD2	C	ASP	61	3.7	H-bond
1	H	PHE	27	O-NH1	C	ARG	64	3.6	H-bond
1	H	PHE	27	CB-O	D	SER	94	4.3	Van-der-Waals
1	H	PHE	27	CB-O	D	LEU	95	3.6	Van-der-Waals
1	H	PHE	27	CB-CA	D	LEU	95	4.1	Hydrophobic
1	H	THR	28	OG1-CZ3	C	TRP	47	4.5	Van-der-Waals
1	H	THR	28	CG2-CD1	C	TYR	58	4.5	Van-der-Waals
1	H	THR	28	OG1-O	C	TYR	59	4.0	H-bond
1	H	THR	28	CG2-NH2	C	ARG	64	3.2	Van-der-Waals
1	H	THR	28	OG1-NH2	C	ARG	64	4.2	H-bond
1	H	THR	28	N-O	D	LEU	95	3.1	H-bond
1	H	THR	28	OG1-O	D	LEU	95	3.5	H-bond
1	H	THR	28	OG1-O	D	SER	95A	2.8	H-bond
1	H	THR	28	OG1-CB	D	ALA	95B	3.7	Van-der-Waals
1	H	THR	28	OG1-N	D	ALA	95B	4.3	H-bond
1	H	PHE	29	N-NH1	C	ARG	64	3.9	Van-der-Waals
1	H	PRO	30	CD-NE	C	ARG	64	3.3	Van-der-Waals
1	H	ALA	31	CB-OG	D	SER	95A	3.7	Van-der-Waals
1	H	PHE	32	CZ-O	D	SER	93	3.8	Van-der-Waals
1	H	PHE	32	CZ-O	D	SER	94	3.4	Van-der-Waals
1	H	PHE	32	CE2-O	D	LEU	95	3.9	Van-der-Waals
1	H	PHE	32	CE2-CB	D	SER	95A	3.5	Van-der-Waals
1	H	ASN	73	O-CD	C	ARG	64	3.9	Van-der-Waals
1	H	SER	74	O-CG	C	ARG	64	3.8	Van-der-Waals
1	H	SER	74	O-CA	C	GLY	65	3.3	Van-der-Waals
1	H	SER	74	O-N	C	GLY	65	3.7	H-bond
1	H	ASN	76	ND2-OD1	C	ASP	61	3.5	H-bond
1	H	ASN	76	OD1-CD	C	ARG	64	3.2	Van-der-Waals
1	H	ASN	76	OD1-NH1	C	ARG	64	3.8	H-bond
1	H	ASN	76	OD1-NE	C	ARG	64	4.1	H-bond
1	H	ARG	94	NH1-O	D	SER	94	2.8	H-bond
1	H	ARG	94	NH2-O	D	SER	94	3.8	H-bond
1	H	ASN	97	ND2-O	D	SER	93	3.6	H-bond
1	H	ASN	97	ND2-OG	D	SER	95A	4.4	H-bond
1	L	SER	56	OG-OG	D	SER	27	2.5	H-bond
2	H	ARG	19	NH1-NH1	O	ARG	19	3.62	Van-der-Waals
2	H	ARG	19	NH1-OE1	O	GLN	81	3.39	H-bond
2	H	ARG	19	NH2-OE1	O	GLN	81	3.44	H-bond
2	H	ASP	72	OD2-NE2	O	HIS	82A	4.34	H-bond
2	H	ARG	75	CD-CE1	O	HIS	82A	3.41	Van-der-Waals
2	H	TYR	79	OH-NE2	O	GLN	81	4.45	H-bond
2	H	TYR	79	OH-CE1	O	HIS	82A	4.32	Van-der-Waals
2	H	GLN	81	OE1-NH1	O	ARG	19	3.38	H-bond
2	H	GLN	81	OE1-NH2	O	ARG	19	3.43	H-bond
2	H	GLN	81	NE2-OH	O	TYR	79	4.44	H-bond
2	H	HIS	82A	NE2-OD2	O	ASP	72	4.32	H-bond
2	H	HIS	82A	CE1-CD	O	ARG	75	3.4	Van-der-Waals
2	H	HIS	82A	CE1-OH	O	TYR	79	4.31	Van-der-Waals
2	H	GLN	81	NE2-OH	O	TYR	79	4.44	H-bond
2	H	HIS	82A	NE2-OD2	O	ASP	72	4.32	H-bond
2	H	HIS	82A	CE1-CD	O	ARG	75	3.4	Van-der-Waals
2	H	HIS	82A	CE1-OH	O	TYR	79	4.31	Van-der-Waals

Supplementary Tables 3-9. Complete set of homotypic contacts identified in both primary and secondary interfaces for all cryo-EM structures in this study.

Table S4. Homotypic contacts in 239-rsCSP cryo-EM structure

Interface	Chain Fab 1	Residue 1	Position 1	Atom	Chain Fab 2	Residue 2	Position 2	Distance (Å)	Predicted interaction
**1**	H	GLN	1	O-O	Q	GLU	64	4.0	Van-der-Waals
**1**	H	GLN	1	N-O	Q	GLY	65	3.0	H-bond
**1**	H	VAL	2	CG2-CA	Q	GLY	65	3.5	Hydrophobic
**1**	H	VAL	2	N-O	Q	GLY	65	4.1	H-bond
**1**	H	VAL	2	CB-CA	Q	GLY	65	4.4	Hydrophobic
**1**	H	ARG	26	O-CA	Q	GLY	65	3.5	Van-der-Waals
**1**	H	ARG	26	O-N	Q	GLY	65	4.1	H-bond
**1**	H	LEU	27	CD1-OE1	Q	GLU	64	3.7	Van-der-Waals
**1**	H	THR	28	CG2-O	Q	LYS	57	3.8	Van-der-Waals
**1**	H	THR	28	OG1-CE1	Q	TYR	59	3.5	Van-der-Waals
**1**	H	THR	28	OG1-OE1	Q	GLU	64	3.5	H-bond
**1**	H	THR	28	N-OE1	Q	GLU	64	3.8	H-bond
**1**	H	ARG	30	NH2-O	Q	SER	55	3.6	H-bond
**1**	H	ARG	30	NE-O	Q	SER	55	4.3	H-bond
**1**	H	ARG	30	NE-OD1	Q	ASN	56	3.1	H-bond
**1**	H	ARG	30	NH2-OD1	Q	ASN	56	4.2	H-bond
**1**	H	ASN	31	ND2-O	Q	LYS	57	2.3	H-bond
**1**	H	ASN	31	ND2-CD1	Q	PHE	58	3.3	Van-der-Waals
**1**	H	PHE	32	CE2-OE1	Q	GLU	64	3.1	Van-der-Waals
**1**	H	PHE	32	CE1-NH2	R	ARG	95A	4.1	Van-der-Waals
**1**	H	LYS	94	NZ-CG	Q	GLU	64	4.4	Van-der-Waals
**1**	H	TRP	96	NE1-OD2	Q	ASP	61	3.0	H-bond
**1**	H	TRP	96	CH2-N	R	ASP	1	3.3	Van-der-Waals
**1**	H	TRP	96	O-NH2	R	ARG	95A	2.6	H-bond
**1**	H	TRP	96	O-NH1	R	ARG	95A	4.3	H-bond
**1**	H	GLY	97	O-O	R	TYR	94	4.2	Van-der-Waals
**1**	H	GLY	97	CA-NH2	R	ARG	95A	3.4	Van-der-Waals
**1**	H	GLY	97	O-NH2	R	ARG	95A	3.6	H-bond
**1**	H	GLY	97	O-NH1	R	ARG	95A	4.5	H-bond
**1**	H	GLY	98	C-O	R	TYR	94	4.4	Van-der-Waals
**1**	H	GLY	98	N-NH2	R	ARG	95A	4.2	Van-der-Waals
**1**	H	ALA	99	CB-NE2	R	GLN	27	3.6	Van-der-Waals
**1**	H	ALA	99	CA-O	R	TYR	94	4.1	Van-der-Waals
**1**	H	ALA	99	N-O	R	TYR	94	4.3	H-bond
**1**	H	ALA	99	CB-OG	R	SER	95	3.3	Van-der-Waals
**1**	H	ALA	99	N-OG	R	SER	95	4.4	H-bond
**1**	H	TYR	102	OH-O	Q	GLU	64	4.0	H-bond
**1**	L	ARG	56	NH1-O	Q	ASP	61	3.1	H-bond
**1**	L	ARG	56	NH2-O	Q	ASP	61	3.8	H-bond
**1**	L	ARG	56	NH1-CA	Q	SER	62	3.2	Van-der-Waals
**1**	L	ARG	56	NH1-O	Q	SER	62	4.0	H-bond
**1**	L	ARG	56	CD-NH2	Q	ARG	83	4.0	Van-der-Waals

Table S5. Homotypic contacts in 311-rsCSP cryo-EM structure

Interface	Chain Fab 1	Residue 1	Position 1	Atom	Chain Fab 2	Residue 2	Position 2	Distance (Å)	Predicted interaction
**1**	H	GLY	26	O-O	Q	GLU	64	4.2	Van-der-Waals
**1**	H	GLY	26	O-CA	Q	GLY	65	3.6	Van-der-Waals
**1**	H	GLY	26	O-N	Q	GLY	65	4.0	H-bond
**1**	H	PHE	27	CB-OE2	Q	GLU	64	4.1	Van-der-Waals
**1**	H	THR	28	OG1-CE1	Q	TYR	59	3.8	Van-der-Waals
**1**	H	THR	28	OG1-OE2	Q	GLU	64	3.5	H-bond
**1**	H	THR	28	N-OE2	Q	GLU	64	3.6	H-bond
**1**	H	SER	30	O-NH2	Q	ARG	56	3.3	H-bond
**1**	H	ASN	31	OD1-NH2	Q	ARG	56	3.1	H-bond
**1**	H	ASN	31	OD1-NE	Q	ARG	56	4.4	H-bond
**1**	H	ASN	31	ND2-O	Q	ASN	57	2.9	H-bond
**1**	H	ASN	31	ND2-CD2	Q	PHE	58	3.7	Van-der-Waals
**1**	H	TYR	32	OH-OE1	Q	GLU	64	3.2	H-bond
**1**	H	TYR	32	OH-OE2	Q	GLU	64	3.4	H-bond
**1**	H	TYR	32	OH-OG	R	SER	95A	4.4	H-bond
**1**	H	TYR	52A	CD1-NH2	Q	ARG	56	4.5	Van-der-Waals
**1**	H	TYR	97	O-O	R	ARG	94	4.2	Van-der-Waals
**1**	H	TYR	98	O-CG	R	ARG	94	3.3	Van-der-Waals
**1**	H	TYR	98	OH-NH1	R	ARG	94	3.4	H-bond
**1**	H	TYR	98	OH-NE	R	ARG	94	4.1	H-bond
**1**	H	TYR	98	OH-NH2	R	ARG	94	4.2	H-bond
**1**	H	TYR	98	O-NE	R	ARG	94	4.2	H-bond
**1**	H	ASP	99	OD2-CB	R	SER	27	4.4	Van-der-Waals
**1**	H	ASP	99	OD2-NH2	R	ARG	93	3.0	Salt-Bridge
**1**	H	ASP	99	OD2-NE	R	ARG	93	4.3	H-bond
**1**	H	ASP	99	OD1-NH2	R	ARG	94	2.5	Salt-Bridge
**1**	H	ASP	99	OD1-NE	R	ARG	94	3.0	H-bond
**1**	H	ASP	99	OD2-NE	R	ARG	94	3.2	H-bond
**1**	H	ASP	99	OD2-NH2	R	ARG	94	3.6	Salt-Bridge
**1**	H	ASP	99	OD1-NH1	R	ARG	94	4.2	Salt-Bridge
**2**	H	ARG	16	NH2-O	X	THR	18	3.5	H-bond
**2**	H	ARG	19	NH2-CB	X	SER	67	3.7	Van-der-Waals
**2**	H	ARG	19	NH1-CB	X	SER	65	4.1	Van-der-Waals
**2**	H	ARG	19	NH2-OG	X	SER	67	4.2	H-bond
**2**	H	ARG	19	NH2-O	X	LYS	66	4.3	H-bond
**2**	H	ARG	19	NH1-OG	X	SER	65	4.4	H-bond

Table S6. Homotypic contacts in 334-rsCSP cryo-EM structure

Interface	Chain Fab 1	Residue 1	Position 1	Atom	Chain Fab 2	Residue 2	Position 2	Distance (Å)	Predicted interaction
**1**	H	ASN	31	OD1-OH	O	TYR	58	2.6	H-bond
**1**	H	ASN	31	ND2-CD	O	LYS	56	4.2	Van-der-Waals
**1**	H	ASN	31	OD1-NZ	O	LYS	56	4.3	H-bond
**1**	H	ASN	31	ND2-OH	O	TYR	58	4.3	H-bond
**1**	H	SER	98	O-CB	P	TYR	94	3.8	Van-der-Waals
**1**	H	SER	99	CB-OG	P	SER	95	3.9	Van-der-Waals
**1**	H	SER	99	CA-O	P	TYR	94	4.1	Van-der-Waals
**1**	H	SER	99	O-OE1	P	GLN	27	4.2	Van-der-Waals
**1**	H	SER	99	O-OG	P	SER	95	4.4	H-bond
**1**	H	SER	100	O-CG	P	TYR	94	3.6	Van-der-Waals
**2**	H	ARG	16	NH1-OD1	T	ASP	17	3.1	Salt-Bridge
**2**	H	ARG	16	CG-CD	T	ARG	18	3.8	Van-der-Waals
**2**	H	ARG	16	NH1-OD2	T	ASP	17	4.4	Salt-Bridge
**2**	H	ARG	16	NH1-O	T	ARG	18	4.5	H-bond
**2**	H	SER	17	O-NE	T	ARG	18	3.0	H-bond
**2**	H	SER	17	OG-OG1	T	THR	20	3.7	H-bond
**2**	H	SER	17	O-NH2	T	ARG	18	3.8	H-bond
**2**	H	LEU	18	CD1-NH2	T	ARG	18	3.4	Van-der-Waals
**2**	H	THR	19	OG1-OG	T	SER	65	3.2	H-bond
**2**	H	THR	19	CG2-OG1	T	THR	74	4.1	Van-der-Waals
**2**	H	THR	68	CG2-CG	T	GLU	70	3.8	Van-der-Waals
**2**	H	SER	70	CB-O	T	SER	67	3.5	Van-der-Waals
**2**	H	SER	70	OG-N	T	SER	67	3.5	H-bond
**2**	H	SER	70	OG-CA	T	GLY	66	3.8	Van-der-Waals
**2**	H	SER	70	OG-O	T	SER	67	3.9	H-bond
**2**	H	ARG	71	O-OG	T	SER	67	3.2	H-bond
**2**	H	ASP	72	OD2-CG2	T	THR	31	3.2	Van-der-Waals
**2**	H	LYS	75	CE-CB	T	SER	52	3.9	Van-der-Waals
**2**	H	TYR	79	CD2-CB	T	SER	65	3.7	Van-der-Waals
**2**	H	TYR	79	OH-OG	T	SER	52	3.9	H-bond
**2**	H	TYR	79	CG-N	T	GLY	66	4.3	Van-der-Waals
**2**	H	GLN	81	NE2-CA	T	GLY	66	3.0	Van-der-Waals
**2**	H	GLN	81	NE2-C	T	SER	65	3.6	Van-der-Waals
**2**	H	GLN	81	NE2-O	T	SER	65	3.7	H-bond
**2**	H	GLN	81	NE2-OG	T	SER	65	3.7	H-bond
**2**	H	GLN	81	CG-OG1	T	THR	72	3.9	Van-der-Waals
**2**	H	GLN	81	OE1-CG	T	GLU	70	4.1	Van-der-Waals
**2**	H	GLN	81	NE2-O	T	GLU	70	4.4	H-bond

Table S7. Homotypic contacts in 337-rsCSP cryo-EM structure

Interface	Chain Fab 1	Residue 1	Position 1	Atom	Chain Fab 2	Residue 2	Position 2	Distance (Å)	Predicted interaction
1	H	SER	28	OG-N	M	LYS	57	3.3	H-bond
1	H	SER	28	OG-O	M	SER	55	3.7	H-bond
1	H	SER	28	OG-O	M	LYS	57	3.8	H-bond
1	H	SER	28	OG-CA	M	LYS	56	3.9	Van-der-Waals
1	H	SER	30	OG-CG	M	LYS	56	3.3	Van-der-Waals
1	H	SER	30	OG-O	M	SER	55	3.3	H-bond
1	H	SER	30	OG-N	M	LYS	56	4.0	H-bond
1	H	SER	30	OG-OG	M	SER	55	4.4	H-bond
1	H	THR	31	CG2-CG	M	LYS	56	3.9	Van-der-Waals
1	H	THR	31	CG2-CZ	M	PHE	58	4.1	Van-der-Waals
1	H	THR	31	CG2-O	M	LYS	57	4.1	Van-der-Waals
1	H	TYR	32	OH-NH2	M	ARG	64	2.5	H-bond
1	H	TYR	32	OH-NH1	M	ARG	64	4.3	H-bond
1	H	HIS	52	CD2-CE	M	LYS	56	4.3	Van-der-Waals
1	H	TYR	99	OH-NH2	M	ARG	64	3.8	H-bond
1	H	TYR	99	CZ-CE2	M	PHE	58	4.0	Hydrophobic
1	H	TYR	99	CE1-CD2	M	PHE	58	4.0	Hydrophobic
1	H	TYR	99	CE1-CE2	M	PHE	58	4.1	Hydrophobic
1	H	TYR	99	CE2-CE2	M	PHE	58	4.2	Hydrophobic
1	H	TYR	99	CZ-CD2	M	PHE	58	4.2	Hydrophobic
1	H	TYR	99	CD1-CD2	M	PHE	58	4.4	Hydrophobic
1	H	TYR	99	CD1-CE2	M	PHE	58	4.4	Hydrophobic
1	H	TYR	99	CD1-CZ2	N	TRP	94	3.8	Hydrophobic
1	H	TYR	99	CD1-CH2	N	TRP	94	3.9	Hydrophobic
1	H	TYR	99	CE1-CH2	N	TRP	94	4.3	Hydrophobic
1	H	PHE	100	CZ-O	N	THR	93	2.8	Van-der-Waals
1	H	PHE	100	CE1-NE1	N	TRP	94	3.2	Van-der-Waals
1	H	PHE	100	CE1-CD1	N	TRP	94	3.6	Hydrophobic
1	H	PHE	100	CE1-CE2	N	TRP	94	3.8	Hydrophobic
1	H	PHE	100	CD1-CD1	N	TRP	94	4.2	Hydrophobic
1	H	PHE	100	CE1-CG	N	TRP	94	4.4	Hydrophobic
1	H	PHE	100	CE1-CZ2	N	TRP	94	4.4	Hydrophobic
1	H	PHE	100	CZ-CD1	N	TRP	94	4.5	Hydrophobic
2	H	ARG	16	NH1-NH1	R	ARG	18	2.9	Van-der-Waals
2	H	ARG	16	NH1-CG2	R	THR	74	3.6	Van-der-Waals
2	H	ARG	16	NH1-OG1	R	THR	74	4.5	H-bond
2	H	SER	17	CB-OG	R	SER	65	3.9	Van-der-Waals
2	H	ARG	19	NE-OG	R	SER	67	2.8	H-bond
2	H	ARG	19	NH2-OG	R	SER	31	2.8	H-bond
2	H	ARG	19	NE-OG	R	SER	31	3.5	H-bond
2	H	ARG	19	NH1-OG	R	SER	31	4.1	H-bond
2	H	ARG	19	NH2-OG	R	SER	67	4.4	H-bond
2	H	ARG	19	CD-O	R	GLY	66	4.5	Van-der-Waals
2	H	GLN	81	NE2-CA	R	GLY	66	3.1	Van-der-Waals
2	H	GLN	81	NE2-N	R	SER	67	3.3	Van-der-Waals
2	H	GLN	81	OE1-OG	R	SER	67	3.9	H-bond
2	H	GLN	81	OE1-N	R	SER	67	4.1	H-bond
2	H	GLN	81	NE2-O	R	GLY	66	4.2	H-bond

Table S8. Homotypic contacts in 356-rsCSP cryo-EM structure

Interface	Chain Fab 1	Residue 1	Position 1	Atom	Chain Fab 2	Residue 2	Position 2	Distance (Å)	Predicted interaction
**1**	H	GLY	26	O-O	Q	GLU	64	4.2	Van-der-Waals
**1**	H	GLY	26	O-CA	Q	GLY	65	3.6	Van-der-Waals
**1**	H	GLY	26	O-N	Q	GLY	65	4.3	H-bond
**1**	H	PHE	27	CB-CG	Q	GLU	64	4.0	Van-der-Waals
**1**	H	PHE	27	CA-CA	Q	GLY	65	4.3	Hydrophobic
**1**	H	THR	28	CG2-O	Q	LYS	57	4.4	Van-der-Waals
**1**	H	THR	28	OG1-CE1	Q	TYR	59	3.2	Van-der-Waals
**1**	H	THR	28	OG1-OE2	Q	GLU	64	2.9	H-bond
**1**	H	THR	28	N-OE2	Q	GLU	64	3.4	H-bond
**1**	H	ARG	30	NH1-O	Q	SER	55	3.6	H-bond
**1**	H	ARG	30	NH2-O	Q	SER	55	4.2	H-bond
**1**	H	ARG	30	NH1-CA	Q	ASN	56	4.1	Van-der-Waals
**1**	H	ARG	30	NH1-OD1	Q	ASN	56	4.2	H-bond
**1**	H	ARG	30	NH2-OD1	Q	ASN	56	4.3	H-bond
**1**	H	ASN	31	ND2-O	Q	LYS	57	2.7	H-bond
**1**	H	ASN	31	OD1-CE2	Q	PHE	58	4.2	Van-der-Waals
**1**	H	PHE	32	CZ-OE1	Q	GLU	64	3.8	Van-der-Waals
**1**	H	LEU	97	O-OD2	Q	ASP	61	3.8	Van-der-Waals
**1**	H	PHE	98	CB-CB	Q	ASP	61	3.7	Van-der-Waals
**1**	H	PHE	98	CE1-N	R	GLN	1	3.8	Van-der-Waals
**1**	H	TYR	99	O-NE2	R	GLN	1	3.1	H-bond
**1**	H	TYR	99	CE2-OD1	R	ASN	93	4.0	Van-der-Waals
**1**	H	ASP	100	OD1-CA	R	GLN	1	3.4	Van-der-Waals
**1**	H	ASP	100	OD1-N	R	GLN	1	4.2	H-bond
**1**	H	ASP	100	OD2-NH2	R	ARG	27	2.7	Salt-Bridge
**1**	H	ASP	100	OD1-NH1	R	ARG	27	2.9	Salt-Bridge
**1**	H	ASP	100	OD1-NH2	R	ARG	27	3.3	Salt-Bridge
**1**	H	ASP	100	OD2-NH1	R	ARG	27	3.6	Salt-Bridge
**1**	H	ASP	100	CB-NH2	R	ARG	27	3.6	Van-der-Waals
**1**	H	ASP	100	OD2-ND2	R	ASN	93	3.8	H-bond
**1**	H	ASP	100	OD1-ND2	R	ASN	93	4.1	H-bond
**1**	H	HIS	100A	N-ND2	R	ASN	93	3.6	Van-der-Waals
**1**	H	HIS	100A	N-OD1	R	ASN	93	3.6	H-bond
**1**	H	ASP	100B	OD2-NH2	R	ARG	27	3.9	Salt-Bridge
**1**	H	ASP	100B	N-ND2	R	ASN	93	4.4	Van-der-Waals
**2**	H	GLN	13	NE2-OE2	X	GLU	17	3.5	H-bond
**2**	H	ARG	16	NH1-CG	X	GLU	17	4.0	Van-der-Waals
**2**	H	ARG	16	NH1-OE1	X	GLU	17	4.1	Salt-Bridge
**2**	H	ARG	16	NH1-O	X	ARG	18	4.1	H-bond
**2**	H	SER	17	O-CD	X	ARG	18	4.5	Van-der-Waals
**2**	H	SER	17	OG-CG2	X	THR	20	3.2	Van-der-Waals
**2**	H	SER	17	OG-OG1	X	THR	20	3.3	H-bond
**2**	H	SER	17	O-OG1	X	THR	20	4.2	H-bond
**2**	H	LEU	18	CD1-NH2	X	ARG	18	4.3	Van-der-Waals
**2**	H	ARG	19	NE-OG	X	SER	65	3.1	H-bond
**2**	H	ARG	19	NH1-OG	X	SER	65	3.6	H-bond
**2**	H	ARG	19	NH2-OG	X	SER	65	3.9	H-bond
**2**	H	ARG	19	NH2-N	X	GLY	66	3.6	Van-der-Waals
**2**	H	ARG	19	NH1-OG1	X	THR	72	4.2	H-bond
**2**	H	LYS	75	NZ-OG	X	SER	52	4.3	H-bond
**2**	H	GLN	81	OE1-OG1	X	THR	72	4.1	H-bond

Table S9. Homotypic contacts in 364-rsCSP cryo-EM structure

Interface	Chain Fab 1	Residue 1	Position 1	Atom	Chain Fab 2	Residue 2	Position 2	Distance (Å)	Predicted interaction
**1**	H	PHE	27	O-OD2	E	ASP	61	3.4	Van-der-Waals
**1**	H	PHE	27	N-OD2	E	ASP	61	4.3	H-bond
**1**	H	PHE	27	C-OD2	F	ASP	1	4.0	Van-der-Waals
**1**	H	THR	28	CA-OD1	E	ASP	61	3.4	Van-der-Waals
**1**	H	THR	28	OG1-OD1	E	ASP	61	3.5	H-bond
**1**	H	THR	28	N-OD2	E	ASP	61	3.9	H-bond
**1**	H	THR	28	N-OD1	E	ASP	61	4.0	H-bond
**1**	H	THR	28	OG1-OD2	E	ASP	61	4.3	H-bond
**1**	H	THR	28	CG2-O	E	TYR	59	4.5	Van-der-Waals
**1**	H	THR	28	OG1-OD2	F	ASP	1	2.4	H-bond
**1**	H	THR	28	OG1-OD1	F	ASP	1	3.0	H-bond
**1**	H	THR	28	N-OD2	F	ASP	1	3.2	H-bond
**1**	H	THR	28	N-OD1	F	ASP	1	3.5	H-bond
**1**	H	THR	28	CG2-CZ	F	TYR	94	3.6	Van-der-Waals
**1**	H	SER	30	OG-NE2	E	GLN	64	3.3	H-bond
**1**	H	SER	30	OG-OE1	E	GLN	64	4.4	H-bond
**1**	H	GLY	31	CA-OH	F	TYR	94	3.4	Van-der-Waals
**1**	H	GLY	31	O-NH2	F	ARG	93	3.5	H-bond
**1**	H	GLY	31	N-OH	F	TYR	94	4.1	H-bond
**1**	H	GLY	31	O-OH	F	TYR	94	4.3	H-bond
**1**	H	GLY	31	CA-CZ	F	TYR	94	4.4	Hydrophobic
**1**	H	TYR	32	OH-NE	F	ARG	93	3.6	H-bond
**1**	H	TYR	32	OH-NH2	F	ARG	93	3.6	H-bond
**1**	H	TYR	32	CE1-OD1	F	ASP	1	3.8	Van-der-Waals
**1**	H	TYR	32	OH-OD1	F	ASP	1	3.9	H-bond
**1**	H	TYR	32	OH-N	F	ASP	1	4.1	H-bond
**1**	H	ASN	73	O-CG	E	GLN	64	4.0	Van-der-Waals
**1**	H	LYS	76	NZ-CG	E	ASP	61	4.2	Van-der-Waals
**1**	H	LYS	76	NZ-OD2	E	ASP	61	4.3	Salt-Bridge
**1**	H	ASP	97	OD2-OE1	F	GLN	27	2.7	Van-der-Waals
**1**	H	ASP	97	OD2-NE2	F	GLN	27	3.3	H-bond
**1**	H	ASP	97	OD1-NE	F	ARG	93	4.1	H-bond

Supplementary Table 10. Summary of biolayer interferometry binding and liver burden data

	$NPNA_4$					$NPNA_8$					rsCSP					
**mAb**	**k_{ON} (1/Ms)**	**k_{OFF} (1/s)**	**K_D1 (nM)**	**K_D2 (nM)**	**K_D avg (nM)**	**k_{ON} (1/Ms)**	**k_{OFF} (1/s)**	**K_D1 (nM)**	**K_D2 (nM)**	**K_D avg (nM)**	**k_{ON} (1/Ms)**	**k_{OFF} (1/s)**	**K_D1 (nM)**	**K_D2 (nM)**	**K_D (nM)**	**Liver burden (% inh.)**
227	1.3E+05	5.8E-03	74.7	0.3	37.5	4.0E+04	3.5E-03	143	17.9	80.3	1.7E+05	2.5E-04	2.5	0.4	1.6	52.5
239	1.1E+05	6.3E-03	56.3	33.1	44.7	8.3E+04	5.6E-04	14.3	0.1	7.2	1.4E+05	6.3E-05	0.6	0.4	0.5	81
239R	4.1E+05	3.3E-02	165	-	165	1.2E+05	2.2E-02	205	-	205.0	3.3E+05	2.9E-02	110	-	110.0	31.2
311	2.7E+04	4.1E-03	353	71.3	212	6.6E+04	2.5E-04	12.1	0.1	6.1	6.6E+04	7.6E-05	2.6	0.8	1.7	87.7
311R	7.1E+04	8.8E-03	131	3.7	67.4	7.1E+04	5.1E-03	401	21.2	211	6.5E+04	5.6E+03	93.8	4.0	48.9	41.4
334	5.3E+04	2.1E-03	55.3	44.5	49.9	5.2E+04	1.3E-03	35.6	9.4	22.4	1.3E+05	1.1E-03	8.2	1.7	4.9	83.6
337	1.3E+05	7.0E-03	45.4	22.3	36.9	6.6E+04	3.5E-03	69.8	9.1	39.4	1.5E+05	1.3E-03	10.8	0.3	5.6	68.4
356	8.0E+04	1.3E-02	185	60.7	123	8.7E+04	5.6E-04	12.6	0.1	6.4	1.4E+05	1.1E-04	1.3	0.3	0.8	87.7
356R	5.6E+05	3.0E-02	132	25.8	79.0	7.9E+04	1.7E-02	245	1.9	124.0	1.6E+05	1.6E-02	102	31.7	67.0	55.9
364	4.3E+04	2.7E-03	512	8.4	260	6.5E+04	2.7E-04	9.7	0.1	4.9	1.3E+05	1.2E-04	0.95	0.92	0.9	85.4
317	8.5E+04	3.0E-04	5.0	2.6	3.8	6.9E+04	1.9E-04	3.1	2.8	2.9	8.3E+05	3.4E-04	0.4	0.3	0.4	91.4

Supplementary Table 10. **Summary of biolayer interferometry binding and liver burden data.** Kinetic parameters are averages across at least 4 fits with $R^2 \geq 0.98$. All binding curves were fit with a 2:1 kinetic model except for 239R, which displayed 1:1 kinetics. K_{ON} and K_{OFF} values are an average of the two association and dissociation constants. BLI was run in duplicate and reported values are an average of the two experiments.

Supplementary Table 11. X-ray data collection and refinement statistics (molecular replacement) for 311R Fab

	311R-(NPNA)3
**Data collection**	
Space group	$P2_12_12_1$
Cell dimensions	
a, *b*, *c* (Å)	45.41, 70.77, 170.25
α, β, γ (°)	90.00, 90.00, 90.00
Resolution (Å)	1.90(1.94-1.90)
R_{sym} or R_{merge}	0.055 (0.625)
$I / \sigma I$	17.2(1.6)
Completeness (%)	99.8(97.6)
Redundancy	3.3(2.3)
**Refinement**	
Resolution (Å)	1.90
No. reflections	44216
R_{work} / R_{free}	0.1839/ 0.2216
No. atoms	3500
Protein	3327
Ligand/ion	
Water	173
B-factors	40.0
Protein	39.91
Ligand/ion	
Water	46.77
R.m.s. deviations	
Bond lengths (Å)	0.0119
Bond angles (°)	1.7183

*Single crystal. *Values in parentheses are for highest-resolution shell.

REFERENCES

1. Oyen, D. et al. Structural basis for antibody recognition of the NANP repeats in Plasmodium falciparum circumsporozoite protein. *Proc Natl Acad Sci U S A* **114**, E10438-E10445 (2017).
2. Pholcharee, T. et al. Diverse Antibody Responses to Conserved Structural Motifs in Plasmodium falciparum Circumsporozoite Protein. *J Mol Biol* **432**, 1048-1063 (2020).
3. Pholcharee, T. et al. Structural and biophysical correlation of anti-NANP antibodies with in vivo protection against P. falciparum. *Nat Commun* **12**, 1063 (2021).
