# Supplementary material for: Affinity-matured homotypic interactions induce spectrum of PfCSP structures that influence protection from malaria infection
Source: Nat Commun. 2023 Jul 28;14:4546. doi: 10.1038/s41467-023-40151-x (PMC10382551; doi:10.1038/s41467-023-40151-x)
Supplement: Supplementary file 3 — Reporting Summary [file 41467_2023_40151_MOESM3_ESM.pdf]

nature portfolio

Corresponding author(s): Ward, A.B.

Last updated by author(s): 06/30/2023

Reporting Summary

Nature Portfolio wishes to improve the reproducibility of the work that we publish. This form provides structure for consistency and transparency in reporting. For further information on Nature Portfolio policies, see our Editorial Policies and the Editorial Policy Checklist.

Statistics

For all statistical analyses, confirm that the following items are present in the figure legend, table legend, main text, or Methods section.

n/a	Confirmed	
☐	☒	The exact sample size (*n*) for each experimental group/condition, given as a discrete number and unit of measurement
☐	☒	A statement on whether measurements were taken from distinct samples or whether the same sample was measured repeatedly
☐	☒	The statistical test(s) used AND whether they are one- or two-sided<br>*Only common tests should be described solely by name; describe more complex techniques in the Methods section.*
☒	☐	A description of all covariates tested
☒	☐	A description of any assumptions or corrections, such as tests of normality and adjustment for multiple comparisons
☐	☒	A full description of the statistical parameters including central tendency (e.g. means) or other basic estimates (e.g. regression coefficient) AND variation (e.g. standard deviation) or associated estimates of uncertainty (e.g. confidence intervals)
☐	☒	For null hypothesis testing, the test statistic (e.g. *F*, *t*, *r*) with confidence intervals, effect sizes, degrees of freedom and *P* value noted<br>*Give P values as exact values whenever suitable.*
☒	☐	For Bayesian analysis, information on the choice of priors and Markov chain Monte Carlo settings
☒	☐	For hierarchical and complex designs, identification of the appropriate level for tests and full reporting of outcomes
☒	☐	Estimates of effect sizes (e.g. Cohen's *d*, Pearson's *r*), indicating how they were calculated

Our web collection on statistics for biologists contains articles on many of the points above.

Software and code

Policy information about availability of computer code

Data collection	Leginon software was used for automated collection of EM data. For crystal structure determination, the datasets were integrated, indexed, and scaled with the HKL2000 package.
Data analysis	Micrograph movie frames were aligned and dose-weighted using MotionCor2. CTF estimation was performed with CTFFind4. Single particle data processing was performed with both cryoSPARC v3 and RELION3.0. For cryo-EM structure modelling, UCSF Chimera, Coot v0.9.8.7, RosettaCM, Rosetta Relax, and PHENIX v20.1.1 were used. X-ray structures were determined with molecular replacement with Phaser, and were refined with Refmac5 and Coot v0.9.8.7. For analysis of biolayer interferometry and liver burden data, GraphPad Prism 9.0 was used.

For manuscripts utilizing custom algorithms or software that are central to the research but not yet described in published literature, software must be made available to editors and reviewers. We strongly encourage code deposition in a community repository (e.g. GitHub). See the Nature Portfolio guidelines for submitting code & software for further information.

Data

Policy information about availability of data

All manuscripts must include a data availability statement. This statement should provide the following information, where applicable:

- Accession codes, unique identifiers, or web links for publicly available datasets
- A description of any restrictions on data availability
- For clinical datasets or third party data, please ensure that the statement adheres to our policy

Cryo-EM structures and density maps generated in this study were deposited to the Protein Data Bank (PDB) and Electron Microscopy Data Bank (EMDB), respectively, with the following accession codes: 227-NPNA8: 8DYT https://doi.org/10.2210/pdb8dyt/pdb, EMD-27781 https://www.ebi.ac.uk/emdb/EMD-27781; 239-rsCSP: 8DYW https://doi.org/10.2210/pdb8dyw/pdb, EMD-27784 https://www.ebi.ac.uk/emdb/EMD-27784; 311-rsCSP: 8DYX https://doi.org/10.2210/pdb8dyx/pdb, EMD-27785 https://www.ebi.ac.uk/emdb/EMD-27785; 334-rsCSP: 8DYY https://doi.org/10.2210/pdb8dyy/pdb, EMD-27786 https://www.ebi.ac.uk/emdb/EMD-27786; 337-rsCSP: 8DZ3 https://doi.org/10.2210/pdb8dz3/pdb, EMD-27787 https://www.ebi.ac.uk/emdb/EMD-27787; 356-rsCSP: 8DZ4 https://doi.org/10.2210/pdb8dz4/pdb, EMD-27788 https://www.ebi.ac.uk/emdb/EMD-27788; 364-rsCSP: 8DZ5 https://doi.org/10.2210/pdb8dz5/pdb, EMD-27789 https://www.ebi.ac.uk/emdb/EMD-27789. The X-ray coordinates for 311R Fab-NPNA3 have been deposited to the PDB under the accession code 8EKF, https://doi.org/10.2210/pdb8ekf/pdb. The crystal structures used in this study for comparison to cryo-EM structures are available in the PDB under the following accession codes. 239-NPNA2: 6W00, https://doi.org/10.2210/pdb6w00/pdb; 356-NPNA2: 6W05, https://doi.org/10.2210/pdb6w05/pdb; 311-NPNA2: 6AXK, https://doi.org/10.2210/pdb6axk/pdb; 364-NPNA2: 6WFW, , https://doi.org/10.2210/pdb6wfw/pdb. Source data are provided with this paper.

Human research participants

Policy information about studies involving human research participants and Sex and Gender in Research.

Reporting on sex and gender	n/a
Population characteristics	n/a
Recruitment	n/a
Ethics oversight	n/a

Note that full information on the approval of the study protocol must also be provided in the manuscript.

Field-specific reporting

Please select the one below that is the best fit for your research. If you are not sure, read the appropriate sections before making your selection.

☒ Life sciences ☐ Behavioural & social sciences ☐ Ecological, evolutionary & environmental sciences

For a reference copy of the document with all sections, see nature.com/documents/nr-reporting-summary-flat.pdf

Life sciences study design

All studies must disclose on these points even when the disclosure is negative.

Sample size	Based on published studies (DOI: 10.1186/s12936-019-3055-9 and DOI: 10.1186/s12936-020-03181-0), the sample size that we used should allow us to define differences between groups in the liver burden experiments.
Data exclusions	No data were excluded from the analysis.
Replication	Biolayer interferometry experiments were performed in duplicate, and produced consistent results across both experiments. Final values are an average of both experiments as explained in Methods. Across the three liver burden experiments in this study, IgG 311 was included in each, while 317 was included in two of three, and both antibodies showed highly consistent levels of protection. Further, many of these same antibodies were previously tested in liver burden studies conducted under near identical conditions, and the results presented here are highly consistent with this work (https://doi.org/10.1038/s41467-021-21221-4). Thus, liver burden experiments were not explicitly replicated. Assessment of in vivo antibody kinetics was not replicated as these data were consistent with previous unpublished data on identical or closely related antibodies. Structure determination was not replicated as the cryo-EM structures were nearly identical to X-ray structures of the same Fabs in complex with peptides (as shown in Suppl. Fig. 2), and the refinement statistics of the final deposited structures were of exceptional quality (Suppl. Table 1).
Randomization	For protection study and in vivo antibody kinetics experiments, all groups of mice were matched by age and sex. Randomization is not relevant for biolayer interferometry and structure determination as these experiments do not involve treatment groups.
Blinding	For in vivo protection (liver burden) and antibody pharmacokinetics experiments, the antibodies were blinded. The investigators doing the

Blinding	mouse experiments received the antibodies coded, which were decoded only after the experiment was completed. Blinding is not relevant to the structural and biophysical experiments as these experiments do not involve treatment groups and are not randomized.

Reporting for specific materials, systems and methods

We require information from authors about some types of materials, experimental systems and methods used in many studies. Here, indicate whether each material, system or method listed is relevant to your study. If you are not sure if a list item applies to your research, read the appropriate section before selecting a response.

Materials & experimental systems

n/a	Involved in the study	
☐	☒	Antibodies
☐	☒	Eukaryotic cell lines
☒	☐	Palaeontology and archaeology
☐	☒	Animals and other organisms
☐	☒	Clinical data
☒	☐	Dual use research of concern

Methods

n/a	Involved in the study	
☒	☐	ChIP-seq
☒	☐	Flow cytometry
☒	☐	MRI-based neuroimaging

Antibodies

Antibodies used	anti-human IgG Fab (Jackson ImmunoResearch 109-006-097)<br>alkaline phosphatase-conjugated goat anti-human IgG Fcγ (Jackson ImmunoResearch 109-005- 008)
Validation	Validation relied on target specificity stated on manufacturer's website, which is as follows. 1. Jackson ImmunoResearch 109-006-097: Based on immunoelectrophoresis and/or ELISA, the antibody reacts with the F(ab')2/Fab portion of human IgG. It also reacts with the light chains of other human immunoglobulins. No antibody was detected against the Fc portion of human IgG or against non-immunoglobulin serum proteins. The antibody has been tested by ELISA and/or solid-phase adsorbed to ensure minimal cross-reaction with bovine, horse, and mouse serum proteins, but it may cross-react with immunoglobulins from other species.<br>2. Jackson ImmunoResearch 109-005- 008: Based on immunoelectrophoresis and/or ELISA, the antibody reacts with the Fc portion of human IgG heavy chain but not with the Fab portion of human IgG. No antibody was detected against human IgM or IgA, or against non-immunoglobulin serum proteins. The antibody may cross-react with immunoglobulins from other species.

Eukaryotic cell lines

Policy information about cell lines and Sex and Gender in Research

Cell line source(s)	ExpiCHO cells – ThermoFisher cat. # A29127
Authentication	Cell lines used were not authenticated.
Mycoplasma contamination	Cell lines are tested monthly for mycoplasma. Results are negative.
Commonly misidentified lines (See ICLAC register)	none

Animals and other research organisms

Policy information about studies involving animals; ARRIVE guidelines recommended for reporting animal research, and Sex and Gender in Research

Laboratory animals	Studies using mice were carried out using 6-8 week old C57BL/6 females, maintained at the animal facilities at Johns Hopkins University Bloomberg School of Public Health and the Scripps Research Institute. Mouse rooms are kept at 40-60% relative humidity at a temperature of 68-79 degrees F, with at least 10 room air changes per hour. Mice have a cycle of 14 hours light and 10 hours darkness.
Wild animals	No wild animals were used in this study.
Reporting on sex	Characterization of the liver burden assay has demonstrated that there are not substantial differences in protective efficacy due to sex. To maintain consistency in this study, however, only female mice were used for liver burden and pharmacokinetics experiments.
Field-collected samples	No fields samples were collected in this study.
Ethics oversight	These studies were performed in strict accordance with the recommendations in the Guide for the Care and Use of Laboratory Animals of the National Institutes of Health. The protocol was approved by the Animal Care and Use Committee of the Johns Hopkins University, protocol number MO18H419.

Note that full information on the approval of the study protocol must also be provided in the manuscript.

Clinical data

Policy information about clinical studies

All manuscripts should comply with the ICMJE guidelines for publication of clinical research and a completed CONSORT checklist must be included with all submissions.

Clinical trial registration	ClinicalTrials.gov identified: NCT01857869
Study protocol	The study protocol can be accessed on https://www.clinicalstudydatarequest.com/ with identifier: 117014. Please refer to the GSK clinical study register.
Data collection	Location: Silver Spring, Maryland, United States, 20910. Study start date: May 20, 2013. Primary completion date: March 24, 2013. Study completion date: December 16, 2014.
Outcomes	Clinical outcomes are reported at clinicaltrials.gov with the identifier provided above. Outcomes are also reported in the following publication: DOI: 10.1093/infdis/jiw237
